# Neural response to monetary incentives in acquired adolescent depression after mild traumatic brain injury: Stage 2 Registered Report

**DOI:** 10.1093/braincomms/fcae250

**Published:** 2024-09-04

**Authors:** Jeremy Hogeveen, Ethan M Campbell, Teagan S Mullins, Cidney R Robertson-Benta, Davin K Quinn, Andrew R Mayer, James F Cavanagh

**Affiliations:** Department of Psychology, University of New Mexico, Albuquerque, NM 87131, USA; Psychology Clinical Neuroscience Center, University of New Mexico, Albuquerque, NM 87131, USA; Department of Psychology, University of New Mexico, Albuquerque, NM 87131, USA; Psychology Clinical Neuroscience Center, University of New Mexico, Albuquerque, NM 87131, USA; Department of Psychology, University of New Mexico, Albuquerque, NM 87131, USA; Psychology Clinical Neuroscience Center, University of New Mexico, Albuquerque, NM 87131, USA; Department of Psychology, University of New Mexico, Albuquerque, NM 87131, USA; Psychology Clinical Neuroscience Center, University of New Mexico, Albuquerque, NM 87131, USA; Department of Psychiatry & Behavioral Sciences, University of New Mexico School of Medicine, Albuquerque, NM 87131, USA; Department of Psychology, University of New Mexico, Albuquerque, NM 87131, USA; Department of Psychiatry & Behavioral Sciences, University of New Mexico School of Medicine, Albuquerque, NM 87131, USA; Department of Neurology, University of New Mexico School of Medicine, Albuquerque, NM 87131, USA; The Mind Research Network/Lovelace Biomedical Research Institute, Albuquerque, NM 87106, USA; Department of Psychology, University of New Mexico, Albuquerque, NM 87131, USA; Psychology Clinical Neuroscience Center, University of New Mexico, Albuquerque, NM 87131, USA

**Keywords:** depression, TBI, adolescence, reward, fMRI

## Abstract

Depression is a common consequence of traumatic brain injury. Separately, spontaneous depression—arising without brain injury—has been linked to abnormal responses in motivational neural circuitry to the anticipation or receipt of rewards. It is unknown if post-injury and spontaneously occurring depression share similar phenotypic profiles. This issue is compounded by the fact that nearly all examinations of these psychiatric sequelae are *post hoc*: there are rarely any prospective assessments of mood and neural functioning before and after a brain injury. In this Stage 2 Registered Report, we used the Adolescent Brain Cognitive Development Consortium dataset to examine if a disruption in functional neural responses to rewards is present in patients with depression after a mild traumatic brain injury. Notably, this study provides an unparalleled opportunity to examine the trajectory of neuropsychiatric symptoms longitudinally within-subjects. This allowed us to isolate mild traumatic brain injury-specific variance independent from pre-existing functioning. Here, we focus on a case-control comparison between 43 youth who experienced a mild traumatic brain injury between MRI visits, and 43 well-matched controls. Contrary to pre-registered predictions (https://osf.io/h5uba/), there was no statistically credible increase in depression in mild traumatic brain injury cases relative to controls. Mild traumatic brain injury was associated with subtle changes in motivational neural circuit recruitment during the anticipation of incentives on the Monetary Incentive Delay paradigm. Specifically, changes in neural recruitment appeared to reflect a failure to deactivate ‘task-negative’ brain regions (ventromedial prefrontal cortex), alongside blunted recruitment of ‘task-positive’ regions (anterior cingulate, anterior insula and caudate), during the anticipation of reward and loss in adolescents following mild brain injuries. Critically, these changes in brain activity were not correlated with depressive symptoms at either visit or depression change scores before and after the brain injury. Increased time since injury was associated with a recovery of cognitive functioning—driven primarily by processing speed differences—but depression did not scale with time since injury. These cognitive changes were also uncorrelated with neural changes after mild traumatic brain injury. This report provides evidence that acquired depression may not be observed as commonly after a mild traumatic brain injury in late childhood and early adolescence, relative to findings in adult cases. Several reasons for these differing findings are considered, including sampling enrichment in retrospective cohort studies, under-reporting of depressive symptoms in parent-report data, and neuroprotective factors in childhood and adolescence.

## Teaser

Contrary to predictions, Hogeveen *et al*. found no evidence of clinically significant depression after adolescent mild traumatic brain injury. Altered brain responses to rewards were linked to mild traumatic brain injury but did not correlate with depression. Depression in youth post-mild traumatic brain injury may not be as significant as in adults.

## Graphical Abstract

**Graphical abstract:**
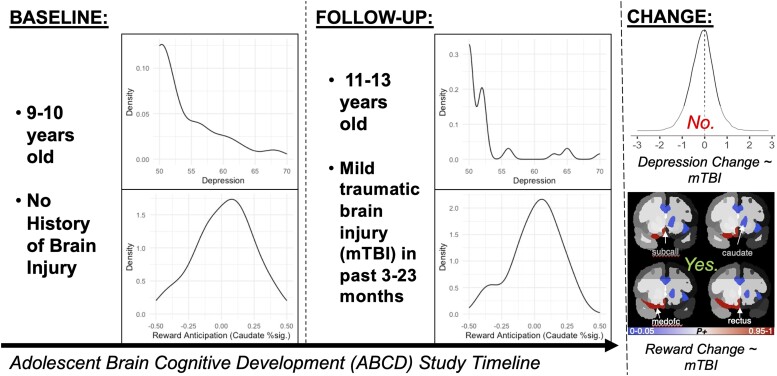

## Introduction

### Background and motivation

Traumatic brain injury (TBI) is a significant global public health problem. Recent decades have seen increased recognition that even *mild* TBI (mTBI) has the potential to cause lasting functional difficulties that can impact the lives of patients and their families for weeks, months or years post-injury.^1^ Depression is one of the most common and persistent functional difficulties experienced by patients in the wake of an mTBI,^2‐4^ and is particularly common in patients with lesions to frontal, temporal and parietal brain networks implicated in emotion and cognitive control.^5,6^ This has led to several recent clinical trials using antidepressant interventions (e.g. repetitive transcranial magnetic stimulation (rTMS), selective serotonin reuptake inhibitors (SSRIs), etc.) to remediate affective and cognitive sequelae of mTBI.^7‐9^ Existing studies of depression in mTBI are often limited by their ability to account for premorbid depressive symptoms. Specifically, the impact of mTBI on depression risk is most often studied cross-sectionally—i.e. comparisons between mTBI patients and control participants are used to provide evidence that depression risk is elevated after an mTBI. There is a central confound in such study designs: mTBI patients with elevated premorbid depression have been shown to report higher levels of lasting post-injury symptoms, which may cause overall inflation of depression and other sequelae of mTBI in cross-sectional studies, independent of the physiological consequences of mTBI.^10‐12^ Additionally, in cases of acquired depression after mTBI, it is unclear whether this occurs as a result of damage to the same neural circuits that underlie mood, anhedonia, or other depressive symptoms absent an mTBI event. Determining whether a single mTBI event can cause increases in depression—and whether acquired depression is driven by similar neural circuit mechanisms to depression without a prior brain injury—are both critical questions that need to be answered to inform evidence-based treatment of depression in mTBI patients.

Emerging results from the *Adolescent Brain Cognitive Development* (*ABCD*^13^) study provide an unparalleled opportunity to address these questions. The *ABCD* study is a large (*N* > 10 000) longitudinal study of children and adolescents in the United States. *ABCD* participants and their parents began the study when participants were 9–10 years old, and a comprehensive battery of cognitive, behavioural, clinical, neuroimaging and other assessments will continue to be administered to these subjects periodically until they reach young adulthood (19–20 years old). There are three specific reasons why *ABCD* can provide crucial insights into the questions addressed in the current Registered Report: (i) depressive symptoms and mTBI history interviews are administered at each study visit, meaning it is possible to determine whether mTBI events trigger lasting changes in depressive symptoms after controlling for premorbid functioning; (ii) multiple gold-standard clinical assays of depression via parent-report are included in *ABCD*, providing a superior assessment of multiple dimensions of the depressive phenotype (e.g. anhedonia, low mood, somatic complaints, etc.) relative to the majority of existing mTBI depression studies; and (iii) the monetary incentive delay (MID) task—a functional neuroimaging task that has been reliably linked to depressive symptoms in adolescence^14‐16^—is being administered at each imaging visit in the *ABCD* study battery.

### *Monetary incentive delay (MID)* task as a functional assay of depression mechanisms

A promising functional neuroimaging task collected during imaging acquisition visits in *ABCD* that has been reliably linked to depression in adolescence is the *Monetary Incentive Delay (MID)* task. The *MID* is an fMRI task commonly used to probe the functional recruitment of motivational brain regions—including the striatum and anterior cingulate cortex (ACC)—during the anticipation and receipt of incentives.^17^ Incentives during the *MID* take the form of monetary gains or losses, each contrasted with a neutral non-reward control condition. This has relevance for depression given the consistent finding that differential behavioural and neural sensitivity to rewards and punishments has been reliably implicated in depression.^18‐22^ In particular, existing *MID*-fMRI studies have identified (i) aberrant ACC and ventral striatal (nucleus accumbens, NAcc) recruitment during incentive anticipation;^20,23,24^ (ii) decreased recruitment of anterior insula (aINS) and brainstem during reward anticipation;^25^ (iii) blunted recruitment of both NAcc and dorsal (caudate) striatum in response to rewarding feedback;^22^ and (iv) decreased caudate-ACC functional connectivity in response to rewarding feedback^26^ in depressed patients. Recruitment of these motivational and prefrontal brain regions in the *MID* is normalized in remitted depression and patients receiving stable doses of escitalopram (serotonin reuptake inhibitor) or amisulpride (dopamine receptor antagonist).^24,26‐29^

Whereas existing *MID*-fMRI studies have primarily recruited adult participants, a key reason this task was included in the *ABCD* protocol is its demonstrated potential to probe the same motivational neural circuitry in adolescent and adult participants. Activation of basal ganglia nuclei (NAcc, caudate, globus pallidus and putamen), ACC, aINS and ventromedial prefrontal cortex has been reliably observed in adolescent participants during reward anticipation and receipt in the *MID* task.^30‐33^ Similar to adult participants, there is strong evidence that recruitment of striatum, aINS, ACC and prefrontal cortex may be aberrant in adolescents with clinically-significant depression and those with an increased familial risk for developing depression in adulthood.^14,15^

In sum, the *MID* task is an established technique for probing the neural bases of diminished reward sensitivity in depression. Aberrant neural circuit recruitment in response to monetary incentives during fMRI is a viable concomitant marker of the depressive phenotype. In this study, we examine this measure for the first time in adolescents before and after a brain injury.

### mTBI studies using the adolescent brain cognitive development (ABCD) study

At each *ABCD* study visit, parents of study participants complete the Ohio State University TBI Identification interview, a well-validated approach for retroactively identifying the frequency and severity of prior brain injury events.^34^ Notably, two early studies have examined the sequelae of mTBI in adolescents via *ABCD*. First, Lopez and colleagues demonstrated that mTBI causes behavioural and emotional problems in adolescents, but found little evidence that brain *structure* was mediating these effects.^35^ Given that most mTBIs are *not* associated with observable abnormalities on standard T1w or T2w MRI scans—apart from cases of ‘complicated mTBI’ cf.^36^—functional MRI assays like the *MID* may be more sensitive predictors of post-mTBI symptom severity than structural imaging. Second, Sheth and colleagues recently demonstrated evidence for increased internalizing and other behavioural problems in adolescents following an mTBI in *ABCD* participants.^37^ Both of these studies provide additional impetus for the current Registered Report—they suggest that emotional symptoms are increased in adolescents following an mTBI. However, the current study will be the first to leverage *ABCD* to determine whether motivational neural circuit recruitment assayed via functional MRI predicts depression symptoms following an mTBI in adolescence.

### Aims of the current registered report

The main objective of the current Registered Report is to determine whether functional recruitment of motivational brain regions is aberrant in adolescents with acquired depression after an mTBI. This will involve three specific hypotheses that we will test using a Pre-registered Study Design (osf.io/h5uba):

**Hypothesis 1:** Chronic mTBI causes new-onset depression in adolescence. This hypothesis will be tested by examining pre-post-mTBI changes in parent-reported depressive symptoms in adolescents in the *ABCD* study.

**Hypothesis 2:** mTBI causes aberrant recruitment of motivational brain regions during the anticipation and receipt of monetary incentives. This hypothesis will be tested by examining pre-post-mTBI changes in responses during both reward and loss conditions on the *MID* task in ACC, aINS and striatum (especially: caudate and NAcc).

**Hypothesis 3:** mTBI-related changes in motivational neural circuitry will be associated with depressive symptoms. Specifically, we hypothesize a negative association, whereby mTBI-related increases in depression symptoms are expected to be associated with mTBI-related decreases in BOLD recruitment during the anticipation and receipt of rewards in the *MID* task.

Our Stage 1 Registered Report can be found in the Supplementary material.

## Methods

### Participants

As of *ABCD* Data Release 4.0 (2021; http://dx.doi.org/10.15154/1523041), *N* = 43 participants experienced an mTBI in between the baseline and 2-year MRI imaging acquisition visits (*N* = 27 reported at year 1 follow-up, and *N* = 16 at year 2). These visits were conducted at one of the 21 study sites across the United States (https://abcdstudy.org/study-sites/) with data collection taking place between September 1, 2016–October 15, 2018. The *ABCD* study was designed and implemented by a large team of scientists who were careful to mitigate potential sources of bias.^38^ This subset from the larger *ABCD* pool was selected because they offer the unique opportunity to examine premorbid-to-post-mTBI changes in depressive symptoms and neural recruitment in adolescence. We also pulled a randomly selected subset of well-matched healthy control participants with no history of possible brain injury from *ABCD*. This was implemented using the software package *MatchIt* to select participants using a nearest neighbor approximation,^39^ matching for key demographic and cognitive variables. Specifically, mTBI participants were matched to a randomly selected group of control participants with respect to handedness, age, race, ethnicity, sex, NIH Toolbox cognition composite scores (at baseline visit), CBCL depression scores (at baseline visit), mean framewise displacement/head motion during the MID scans and socioeconomic status (indexed using the Area Deprivation Index, ADI^40^). Given this was limited to participants with available data for all matching variables, the final sample of mTBI and control participants to be included in our analysis is a case-control matched subset of the larger *ABCD* cohort including *N* = 43 mTBI and *N* = 43 control participants (Table 1).

**Table 1:** Demographic and other key matching variables between mTBI and control participants

Variable	Control Participants	mTBI Participants
N	43	43
Female (N)	13	14
Female (proportion)	0.30	0.33
Age (minimum, in months)	108	109
Age (SE, in months)	1.63	2.12
Caucasian (N)	36	35
Caucasian (proportion)	0.84	0.81
Hispanic (N)	10	10
Hispanic (proportion)	0.23	0.23
ADI (mean)	93.64	92.18
ADI (SE)	2.63	3.75
NIH Toolbox Composite (mean)	103.39	103.54
NIH Toolbox Composite (SE)	1.74	1.21
CBCL Depression (mean)	53.60	53.63
CBCL Depression (SE)	0.73	0.78
Head motion (Mean FD)	0.33	0.30
Head motion (SE FD)	0.05	0.04

### Sample size considerations

As there are no prior studies that examine pre-post mTBI neural recruitment within-subjects in a task fMRI paradigm in adolescence, we were forced to compare the proposed sample size to prior studies using cross-sectional comparisons at a single time-point. From this review of the literature, the proposed sample is over three times the size of existing studies (e.g. *N* = 12 mTBI versus control^41^; *N* = 8 versus 8^42^; and *N* = 11 versus 11^43^). Also notably, our within-subjects design looked at pre-post injury changes in symptoms, which is by definition a more powerful design than these cross-sectional studies.^44^ We also used the MID task which yields robust and large effect size fMRI responses to reward, detectable in small samples (*N* = 6 needed for 0.80 power^45^). Finally, we were limited in terms of our proposed power as this was a secondary data analysis rather than a *de novo* data collection effort. Accordingly: Given the importance of the research question, the uniqueness of the dataset (i.e. ability to look at pre-post mTBI change within-subjects), and the fact that the sample is more than 3* the size of existing cross-sectional studies in this field—it was clinically and scientifically imperative to carry out this work.

### Procedure

#### Brain injury identification

The *ABCD* study uses the Ohio State Brain Injury identification method,^34^ administered both at baseline and each follow-up visit. For follow-up visits, the parent or guardian of each *ABCD* participant is asked eight questions, each involving follow-up responses:

1. Since we last saw you on [previous interview date], has your child been hospitalized or treated in an emergency room following an injury to their head or neck?
  1.1. (If 7 = ‘Yes’) Was your child knocked out or did they lose consciousness (LOC)? If yes, how long?
  1.2. (If 7 = ‘Yes’) Was your child dazed or did they have a gap in their memory from the injury?
  1.3. (If 7 = ‘Yes’) How old was your child?
2. Since we last saw you on [previous interview date], has your child injured their head or neck in a car accident or from crashing some other moving vehicle like a bicycle, motorcycle, or ATV? (If yes, 2.1–2.3 same as 1.1–1.3)
3. Since we last saw you on [previous interview date], has your child injured their head or neck in a fall or from being hit by something? (For example, falling from a bike or horse, rollerblading, falling on ice, being hit by a rock). In the past year, has your child injured their head or neck playing sports or on the playground? (If yes, 3.1–3.3 same as 1.1–1.3)
4. Since we last saw you on [previous interview date], has your child injured their head or neck in a fight, from being hit by something, or from being shaken violently? In the past year, has your child been shot in the head? (If yes, 4.1–4.3 same as 1.1–1.3)
5. Since we last saw you on [previous interview date], has your child been nearby when an explosion or blast has occurred? (If yes, 5.1–5.3 same as 1.1–1.3)
6. Do you want to report any more injuries with loss of consciousness since we last saw you on [previous interview date]? (If yes, 6.1–6.3 same as 1.1–1.3)
7. Since we last saw you on [previous interview date], did your child experience a period of time when they experienced multiple, repeated impacts to the head? (e.g. abused, contact sports)?
  7.1. Was your child knocked out or did they lose consciousness (LOC)? If yes, how long?
  7.2. Was your child dazed or did they have a gap in their memory from the injury?
  7.3. At what age did these effects begin?
  7.4. At what age did these effects end?
8. The next question asks about concussions. A concussion is when a blow or jolt to the head causes problems such as headaches, dizziness, being dazed or confused, difficulty remembering or concentrating, vomiting, blurred vision, or being knocked out. Since we last saw you on [previous interview date], how many times did your child have a concussion from playing a sport or being physically active?
  8.1. (If 8 = 1) Approximately how many days of school did your child miss, if any, due to the effects of the concussion?
  8.2. (If 8 > 1) Thinking about the most serious concussion your child experienced since we last saw you [previous interview date], approximately how many days of school did your child miss, if any, due to the effects of the concussion?

TBI severity from these data was then determined according to the following spectrum: (i) improbable TBI (no TBI or TBI without loss of consciousness or memory loss); (ii) possible mTBI (TBI without loss of consciousness, but with memory loss); (iii) mTBI (TBI with ≤30 minute loss of consciousness); (iv) moderate TBI (TBI with loss of consciousness 30 minutes >24 hours); and (v) severe TBI (TBI with loss of consciousness ≥24 hours). Participants were also classified as to whether or not they experienced repeated TBI events. Given our aims, we focused exclusively on participants with a classic mTBI (i.e. including loss of consciousness ≤30 minutes), and only a single isolated mTBI event between the baseline (9–10 years) and 2-year-follow-up (11–12 years old) ABCD study visits.

#### Depression

Clinical depressive symptoms were assessed using a combination of the Child Behavior Checklist (*CBCL*) via the Achenbach System for Empirically-Based Assessment^46^ and a computerized administration of the Kiddie Schedule for Affective Disorders and Schizophrenia diagnostic tool *K-SADS*.^47^ The CBCL is a 112-item parent-administered assay of a broad range of psychological and behavioural problems in youth. Given the hypotheses of the current study, we focused on the DSM-oriented composite score for depressive symptoms from the *CBCL*, which assays symptoms of Major Depression and Dysthymia. Additionally, given our specific interest in reward processing aberrations in depression, we examined the impact of mTBI on present anhedonia ratings based on retrospective reports in the *K-SADS*. Despite the presence of K-SADS results at baseline and at least one follow-up visit for all of our samples, clinical anhedonia was rarely reported. There were only four reports of parent-reported anhedonia (*N* = 2 mTBI; *N* = 2 control), making inferential analyses of anhedonia in the current dataset untenable. The *K-SADS* administered in the *ABCD* study comprises parent-report, which demonstrates high concordance with clinician interview versions of the *K-SADS*.^48^ We opted to rely on parent-report as there are no research-reliable clinician assessments in *ABCD*, and parent-report tends to map better onto clinician ratings of internalizing in adolescent participants compared to self-report.^49^ This is a limitation of the current design and one that should be followed up in future prospective youth mTBI studies that include clinician or other expert (e.g. teacher) reports of internalizing and externalizing problems to examine concordance across reporters.^50^

#### Monetary incentive delay (MID) task

**Figure 1:**
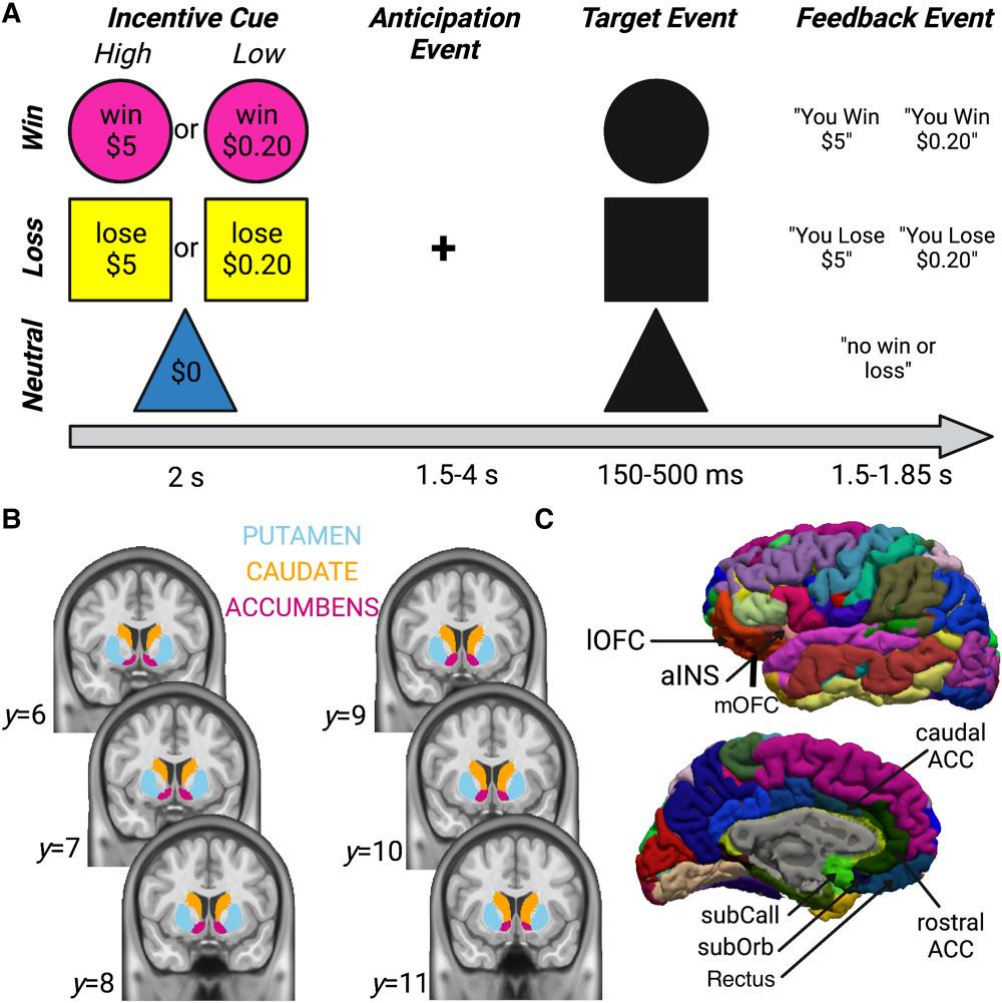
**Behavioural task design and *a priori* regions-of-interest**. (**A**) Schematic of the *ABCD* study’s *Monetary Incentive Delay (MID)* task, adapted from Casey *et al*.^51^ Note that target duration is titrated based on individual reaction times to ensure ≈60% accuracy. (**B**) Subcortical regions-of-interest (ROIs) comprised the putamen, caudate, and nucleus accumbens, and (**C**) the cortical ROIs comprised medial and lateral parcels including lateral orbitofrontal cortex (lOFC), the short gyrus of the insula (anterior insula, aINS), caudal and rostral parcels of the anterior cingulate cortex (caudal ACC and rostral ACC), subcallosal gyrus (subCall), both suborbital and medial sectors of OFC (subOrb and mOFC), and gyrus rectus (Rectus). Both subcortical segmentations and cortical parcellations in the *ABCD* study are generated using subject-specific surface reconstructions in the *Freesurfer* toolbox.^52^

The *MID* task is a widely used method for probing motivational neural circuit recruitment during the anticipation and receipt of positive (monetary gain) or negative (monetary loss) feedback (Fig. 1A^17,30^). In the *ABCD* study version of the *MID* task, each trial begins with a 2 sec long incentive cue that indicates one of five trial types: ‘Win $0.20,’ ‘Win $5,’ ‘Lose $0.20,’ ‘Lose $5,’ and ‘$0.’ This is followed by a fixation cross that is jittered between 1.5 and 4 sec to optimize blood oxygenation-level dependent (BOLD) signal deconvolution during incentive anticipation—i.e. the ‘anticipation event’ in Fig. 1A. After the anticipation phase, a target is presented for 150–500 ms, and participants are required to press a button as quickly as possible before the target disappears. The duration of target events are titrated to ensure that subjects are able to press the button before it disappears on ≈60% of trials. Lastly, the target is followed by a feedback event that tells the participant whether they have successfully received the reward (‘Win’ conditions), avoided the loss (‘Lose’ conditions), or that neither a win or loss has occurred (‘$0’ neutral condition). The current study analyzed six key event-related BOLD contrasts of interest from the MID task: (i–ii) anticipated incentive versus neutral (i.e. win versus neutral, and loss versus neutral); (iii–iv) anticipated high versus low incentive (i.e. win high versus low, and loss high versus low); and (v–vi) positive versus negative feedback (i.e. win versus no win, and no loss versus loss).

#### Magnetic resonance imaging (MRI)

The *ABCD* MRI acquisition and analysis protocols are elaborated in considerable detail elsewhere.^51,53^ Briefly, MRI acquisition is taking place at 21 sites across the United States on 3T MRI scanners. The *ABCD* imaging team has harmonized the image acquisition sequences across all site scanners, but regardless study site was included as a random effect in all inferential analyses in the current study. Youth in the study complete motion-compliance training prior to their MRI scanning sessions in a mock MRI environment, and additional Framewise Integrated Real-Time MRI Monitoring Software FIRMM^54^ is being used to monitor head motion in real time, enabling the scanning team to correct motion online by providing verbal feedback to participants or to collect additional data.^53^ A robust fMRI preprocessing and analysis pipeline that is developed and maintained by the *ABCD* Data Analysis and Informatics Center—the Multi-Modal Processing Stream^53^—is applied to all *ABCD* study data. This stream includes extensive within-subjects head motion de-contamination, including standard realignment parameters, their derivatives and censoring of high motion timepoints.^55,56^ We also included mean framewise displacement as a matching parameter to eliminate the potential for between-group differences in head motion to confound our primary analyses (Table 1). This processing stream also includes quality control of each post-processed structural and functional MRI scan by a team of trained research technicians.^53^ All processed data and tabulated region of interest (ROI) based analysis results are then made publicly available via the National Institute for Mental Health (NIMH) Data Archive (NDA). Subcortical ROI estimates in the publicly-released tabulated imaging data are segmented using the standard *Freesurfer* aseg atlas,^52^ and cortical estimates are parcellated using the two standard *Freesurfer* aparc atlases (Desikan^57^; Destrieux^58^). Here, we used the Destrieux atlas given its higher parcel count and potential for greater functional specificity. Our specific ROIs of interest were: ACC, aINS and striatum (Fig. 1B and C). There are some notable limitations of this type of ROI-based approach (e.g. restricted concordance between ROIs and actual cluster-level activation patterns not always robust^59^). But notably, ROI-based approaches have also been argued to boost model efficiency and demonstrate a reduced tendency to overrepresent large cortical parcels close to the surface relative to traditional whole brain familywise error-corrected inferences—alongside greater sensitivity to smaller subcortical and midline brain regions that are the focus of the current analyses.^60^

### Data analysis plan

Data analyses were conducted using a combination of *Python v.3* and *R v.4* programming languages, and all code are publicly available on the Open Science Framework website (https://osf.io/h5uba/). Therefore, the current results can be recreated by any researchers with authorization to download the *ABCD* Data Release 4.0.

#### Analysis plan for hypothesis 1

We tested the hypothesis that a single mTBI event causes increased post-injury depressive symptoms in adolescents. This hypothesis was tested in a series of two Bayesian Multilevel Models using *brms v.2*. The first model (*Model 1*) used pre-post-mTBI changes in *CBCL* DSM-module depression scores as the outcome variable:


$$\begin{array}{rl} Y\;= & CBCL\;\;depression\;\;after\;\;mTBI\\ & -\;CBCL\;\;depression\;\;at\;\;baseline \end{array}$$

The *Y* response distribution in *Model 1* was fit as a Student *t*-distribution with non-informative priors set at *mu* = 0 and *sigma* = 1. This is intended to accommodate extreme, potentially influential observations at the tail ends of the distribution better than a Gaussian alternative, which will be verified by posterior predictive checks contrasting Student and Gaussian model fits. The model took the form:


Y∼1+TBI+(1|site)

In other words, we modelled pre-post-mTBI changes in *CBCL* depression as a function of the fixed effect of *TBI* (1 = yes, 0 = no). In *Model 1*, a posterior distribution providing credible evidence that the TBI term is above 0 [i.e. median estimate and lower and upper bounds of the 95% high-density interval (HDI) > 0] was taken as evidence for the alternative hypothesis: That mTBI status credibly increases *CBCL* depression scores.

In our preregistered analysis plan, *Model 2* was planned to be identical to *Model 1*, except the *Y* variable was pre-post-mTBI changes in *K-SADS* ratings of anhedonia. However, after inspecting the data for this *Stage 2* Report only *N* = 2 mTBI participants and *N* = 2 control participants had this clinical symptom present at the 2-year-follow-up visit. Therefore, we did not have sufficient cases of acquired anhedonia to run this analysis and *Model 2* was not included in the current manuscript.

#### Analysis plan for hypothesis 2

The modelling approach for the *MID* task fMRI data was similar to our approach for depression scores, but with some slight variations to enable us to simultaneously model all cortical and subcortical ROIs listed in Fig. 1B and C cf.^60‐62^ Specifically, for each ROI we computed the pre-post-mTBI difference score in BOLD responses, and fit separate Bayesian multilevel models for each *MID* task fMRI contrast (6 fMRI *Y* variables):


$$\begin{array}{rl} & Model\;3:Y1\\ & \;=\;anticipate\;\;win\;\;versus\;\;neutral\;\;after\;\;mTBI\\ & \;\;-\;before\;\;mTBI \end{array}$$

$$\begin{array}{rl} & Model\;4:Y2\\ & \;=\;anticipate\;\;loss\;\;versus\;\;neutral\;\;after\;\;mTBI\\ & \;\;-before\;\;mTBI \end{array}$$

$$\begin{array}{rl} & Model\;5:Y3\\ & =\;anticipate\;\;high\;\;versus\;\;low\;\;win\;\;after\;\;mTBI\\ & \;\;-\;before\;\;mTBI \end{array}$$

$$\begin{array}{rl} & Model\;6:Y4\\ & =\;anticipate\;\;high\;\;versus\;\;low\;\;loss\;\;after\;\;mTBI\\ & \;\;-before\;\;mTBI \end{array}$$

$$\begin{array}{rl} & Model\;7:Y5\\ & =\;positive\;\;feedback\;\;win\;\;versus\;\;no\;\;win\;\;after\;\;mTBI\\ & \;-\;before\;\;mTBI \end{array}$$

$$\begin{array}{rl} & Model\;8:Y6\\ & =\;positive\;\;feedback\;\;no\;\;loss\;\;versus\;\;loss\;\;after\;\;mTBI\\ & \;-\;before\;\;mTBI \end{array}$$

Similar to *Model 1*, in *Models 3–8* the *Y* response distribution was fit as a non-informative Student t-distribution centered at 0, and posterior predictive checks were run to ensure that this was appropriate relative to a Gaussian. These models took the form:


Y∼1+TBI+(TBI|ROI)+(1|site:subject)

Given that we had multiple observations for each study subject (i.e. multiple ROI estimates) in these models, in *Models 3–8* we fit the nested random effect of the subject within the study site. We examined the posterior estimates for each ROI (i.e. each subcortical segment or cortical parcel in Fig. 1B and C) in *Models 3–8* and determined how they were modulated by the fixed effect of mTBI status [i.e. (TBI|ROI)]. Given that pre-post-injury changes in cognitive functioning are a common feature of the mTBI phenotype—and may inject unwanted random variation on the inferences in Models 3–8 (e.g. due to reduced vigilance during the task)—we also examined pre-post-mTBI changes in NIH Toolbox Cognition composite scores and determined whether they were correlated with any mTBI-related functional brain changes observed in *Model 3–8*.

Based on prior links to depression, we hypothesized that the BOLD evoked responses during reward versus neutral anticipation, anticipation of high versus low rewards, and the receipt of rewarding versus non-rewarding feedback would be negatively impacted by mTBI. In other words, we expected that some (primarily: NAcc and ACC) or all of the *a priori* subcortical and cortical ROIs would demonstrate **negative** modulation as a function of mTBI status in *Model 3*, *Model 5* and *Model 7*. Importantly, the main output from each *brms* model was a joint probability distribution across participants and ROIs, therefore multiple comparisons at the ROI-level are not appropriate or necessary.^61‐63^ That said, given we examined a total of *N* = 3 win-related contrasts across our primary depression-related fMRI models of interest, we adjusted the size of the posterior probability distribution that we would typically use to judge statistical credibility in Bayesian hierarchical models from 0.15 to 0.05 (i.e. 0.15/3 = 0.05). To be clear: For our fMRI models, posterior distributions where 95% of posterior MCMC estimates were above 0 would be used to indicate a statistically credible increase in BOLD activity as a function of mTBI, whereas those with 5% above 0 would indicate credible evidence for a decrease in BOLD activity as a function of mTBI).

#### Analysis plan for hypothesis 3

We determined whether mTBI-related changes in neural recruitment during the anticipation and receipt of rewards were tied to mTBI-related changes in depression. This series of models (*Models 9–11*) were performed on the same *Y* outcome variables as *Model 3, Model 5* and *Model 7*, with the data filtered to only mTBI participants. These models took the form:


Model9:Y1∼1+CBCLchange+(CBCLchange|ROI)+(1|site:subject)

Model10:Y3∼1+CBCLchange+(CBCLchange|ROI)+(1|site:subject)

Model11:Y5∼1+CBCLchange+(CBCLchange|ROI)+(1|site:subject)

Where *CBCL change* represents the pre-post-mTBI depression score change. We hypothesized that mTBI-related blunting of neural recruitment during the *MID* task would be associated with mTBI-related increases in depression. Therefore, any ROIs where the change in BOLD recruitment was negatively modulated as a function of changes in depression scores (i.e. blunted *MID* task BOLD signal post-mTBI associated with increased *CBCL Depression* scores post-mTBI) would provide credible evidence for hypothesis 3. For any credible effects of mTBI in models 9–11, we would also add *time since injury (in months)* as a potential covariate to determine whether the incremental validity of depression for predicting MID task neural recruitment remained statistically credible in mTBI after covarying for the amount of time that elapsed between the brain injury event and the follow-up fMRI visit. Similar to Models 3, 5 and 7, for Models 9–11 statistical credibility was evaluated based on whether the median and 95%-HDI estimates from the posterior distributions for each ROI are all *below* zero.

#### Robustness and convergence checks

We conducted posterior predictive checks on all of our behavioural (CBCL, NIH Toolbox) and fMRI (MID task fMRI) models and determined that the proposed Student’s *t* non-informative priors were superior to Gaussian distributions for fitting both the central and extreme values in each outcome variable (see POSTERIOR PREDICTIVE CHECKS sections of abcd_mTBI_depression_RR_2023_Stage2.html on osf.io/h5uba). All *brms* models used 4 Markov Chain Monte Carlo (mcmc) chains with 10 000 iterations per chain, and the convergence criterion was *R* < 1.1. To minimize the likelihood of biased posterior samples with divergent transitions or tree depth exceptions, the *adapt_delta* parameter for each model was set to 0.99 and *max_treedepth* set to 15. These parameters were generally robust and nearly all models converged, but in rare instances where a model was flagged with effective sample size or other convergence warnings the data were re-modelled using 20 000 iterations per chain.

#### Exploratory analyses

We conducted additional exploratory analyses to determine whether mTBI is associated with other psychiatric and cognitive sequelae in the current sample.

##### Anxiety

We repeated the analysis plan outlined for mTBI-related changes in *CBCL* depression scores, but including *CBCL* DSM-module anxiety scores. Akin to depression, acquired anxiety is also a clinically-significant consideration after an mTBI.^64‐66^ Existing studies have linked anxiety to aberrant neural recruitment during the anticipation and receipt of incentives during the *MID* task, with studies consistently finding aberrant loss-evoked BOLD recruitment in individuals with various anxiety disorder phenotypes.^67‐69^ We, therefore, hypothesized that ROIs where loss-related neural recruitment is impacted by mTBI in *Model 4*, *Model 6* and *Model 8* would be associated with increased post-mTBI changes in anxiety symptoms.

##### Cognitive performance

A significant proportion of youth recover from post-mTBI sequelae within the first 1-year post-injury.^70,71^ Previous studies have suggested that a return to baseline depression levels after mTBI can be associated with concomitant resolution of cognitive deficits.^4,72^ Accordingly, we analyzed pre-post-mTBI changes in cognitive functioning measures from the *NIH Toolbox* battery.^73^ We anticipated that mTBI subjects would demonstrate impaired performance on the *NIH Toolbox* cognition battery. Conversely, if chronic mTBI patients in the current study did not demonstrate marked increases in depression, we hypothesized that they may also demonstrate similar performance on the *Toolbox* across visits. Additionally, we examined the relationship between time since injury and *NIH Toolbox* battery scores.

##### Repetitive mTBI versus single mTBI

**Figure 2:**
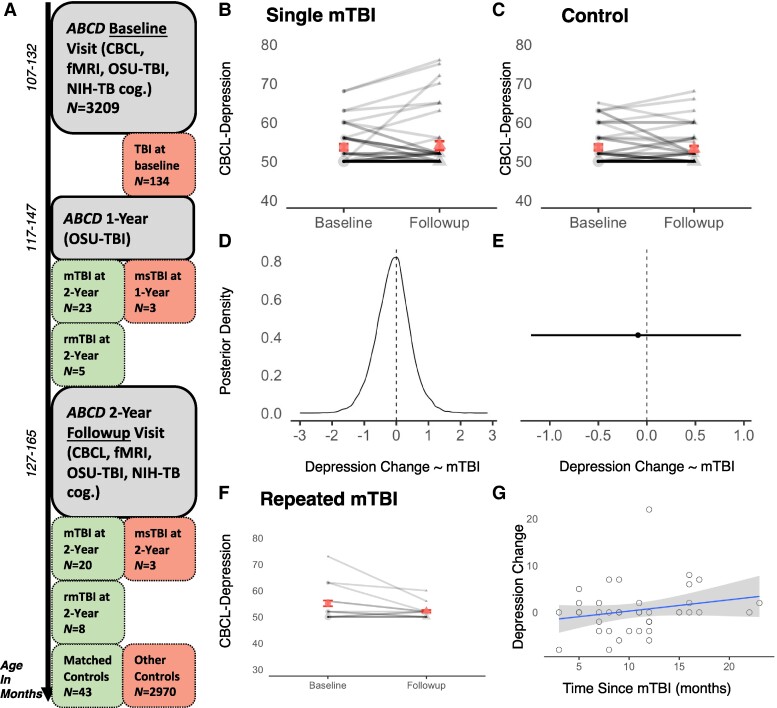
**Study overview and depression results**. (**A**) Participants with all required data points for the pre-registered matching procedure were pulled from the *ABCD* dataset (*N* = 3209). *N* = 134 had some TBI history at baseline and were excluded. *N* = 6 experienced a moderate-to-severe TBI (msTBI) between study visits and were also excluded from our planned analysis. *N* = 43 participants had an mTBI event at either the 1-year or 2-year *ABCD* follow-up visits, and *N* = 13 had repeated mTBIs. These subjects were included in various analyses. Lastly, *N* = 43 controls with no history of TBI were randomly sampled to closely match the mTBI group. Light red indicates exclusion, light green indicates inclusion. (**B, C**) CBCL depression did not differ between baseline and 2-year-follow-up for either mTBI (*d* = −0.18, *95%-high density interval (HDI)* = −0.96 to 0.55) or non-brain-injured controls (*d* = 0.03, *95%-HDI* = −0.64 to 0.65). These inferences involved Bayesian estimation-based analogues of the standard within-subjects *t*-test. Accordingly, the inferences included in the figure caption represent a Bayesian equivalent of Cohen’s d for the effect size of the posterior distribution comparison, as well as they 95% high density interval (HDI) for the distribution of differences between those posteriors. (**D**) *Model 1* posterior distribution of CBCL change scores as a function of mTBI status, and (**E**) mTBI cofficient and error estimate, did not provide credible evidence for a change in depression pre-post injury in mTBI. (**F**) Repeated mTBI events was also not associated with an increase in depression (*d* = 0.45, *95%-HDI* = −0.48 to 1.3). (**G**) Association between time since injury and depression change scores was not statistically credible in mTBI, and trended in the opposite direction than hypothesized (i.e. increase in depression as time since injury increased; *rho* = 0.21, *95%-HDI* = −0.10 to 0.50). These inferences involved Bayesian estimation-based analogues of the standard rank-ordered bivariate correlation test. Accordingly, the inferences included in the figure caption represent a Bayesian equivalent of Spearman’s rho for the effect size of the associations, as well as they 95% high density interval for the distribution of differences between those posteriors.

The current Report was primarily designed to assess the impact of a *single* mTBI event on adolescent depression and task-related fMRI responses to incentives. Prior studies have provided evidence that repetitive versus single mTBI events may be more likely to trigger lasting acquired depression and changes in neural recruitment.^6,74,75^  *N* = 13 participants in the *ABCD* data release 4.0 (2021) experienced multiple mTBIs between baseline and follow-up study visits (Fig. 2A). Therefore, to explore whether repeated mTBI events cause increased depression symptoms or aberrant neural recruitment relative to a single mTBI event—we will repeat our proposed series of analyses testing hypotheses 1–3 but directly contrasting mTBI participants with a single versus 2–3 mTBI events between study visits.

## Results

### Pre-Registered analyses

#### Does mTBI cause new-onset depression in adolescence?

##### Single mTBI versus controls

We first examined changes in depression between baseline and 2-year-follow-up for participants who *did* versus *did not* have an mTBI between *ABCD* study visits. Neither mTBI (*Baseline mean* = 53.6, *sem* = 0.78; *Follow-up mean* = 54.0, *sem* = 1.11; *difference d* = −0.18, *95%-HDI* = −0.96 to 0.55) nor the non-brain-injured control participants (*Baseline mean* = 53.6, *sem* = 0.73; *Follow-up mean* = 53.2, *sem* = 0.71; *difference d* = 0.03, *95%-HDI* = −0.64 to 0.65) demonstrated a statistically credible change in depression across visits (Fig. 2A and B). Additionally, our pre-registered *Model 1* analysis did not indicate evidence that the depression change scores in mTBI were credibly different relative to controls (*b* = −0.10, *se* = 0.53, *95%-CI* = −1.19 to 0.97; Fig. 2C and D).

##### Repeated mTBI and time since injury

To follow directly from *Model 1*, we determined whether repeated mTBI or a shorter time since the mTBI event are associated with increased depression in the ABCD sample. First, we found that *N* = 13 participants that had experienced more than one mTBI event also did not demonstrate a change in depression pre-post injury (*Baseline mean* = 55.2, *sem* = 1.97; *Follow-up mean* = 52.2, *sem* = 0.80; *difference d* = 0.44, *95%-HDI* = −0.48 to 1.3; Fig. 2E). Additionally—after determining that the range of time since injury in the single mTBI group comprised the early-to-late chronic period (i.e. 3- to 23-months; *mean* = 10.5 months, *sd* = 4.63)—we did not observe any evidence for time-dependent changes in post-injury depression. In fact, the numerical relationship was in the opposite direction from our *a priori* predictions (*rho* = 0.20, *95%-HDI* = −0.10 to 0.51; Fig. 2F). Therefore, across several analyses, we failed to observe evidence for acquired depression in adolescence after a single or repeated mTBI in participants 3–23 months post-injury.

##### A note regarding ‘non-brain-injured controls’

As described in the **Participants** section, we used *MatchIt* to identify a group of non-brain-injured control participants that were identical to our mTBI group with respect to demographic variables and premorbid functioning. To be clear, this control group had not necessarily experienced medical trauma between study visits according to the *ABCD* medical history questionnaire. It has been suggested that a different type of emergency injury event—e.g. a broken bone—may represent a better comparison for studying the mental health sequelae of mTBI. However, the current data found that youth with mTBIs did not show increased depression after the injury relative to this uninjured control group. This provides strong support for the null hypothesis—that mTBI did not cause an increase in depression above and beyond normal fluctuations in mood over the same developmental period in the current dataset.

#### Does mTBI cause aberrant recruitment of motivational brain regions during the anticipation and receipt of monetary incentives?

##### Overview of modelling approach

We modelled BOLD responses during each primary contrast from the MID task in 22 *a priori* ROIs in a two-step procedure. In step 1, we collapsed across subjects, groups and study visits, but including random intercepts for ROI and study site (i.e. the latter to account for scanner-specific effects). We then examined the posterior distribution of each random ROI intercept (henceforth, *ROI effects*). In step 2, we ran our preregistered *Models* (*3–8*; see **Methods**) which yielded both a Baseline-Follow-up random intercept for each ROI (henceforth, *ROI change effect*) as well as the random slope associated with mTBI status for each ROI in the model (henceforth, *mTBI change effect*). The step 1 model was used to confirm that motivational neural circuits implicated in the MID task in existing studies were also recruited in this sample. The step 2 model tested our central hypotheses about whether changes in motivational neural circuit recruitment over time is modulated by mTBI status in late childhood/early adolescence.

##### Reward-related fMRI contrasts

**Figure 3:**
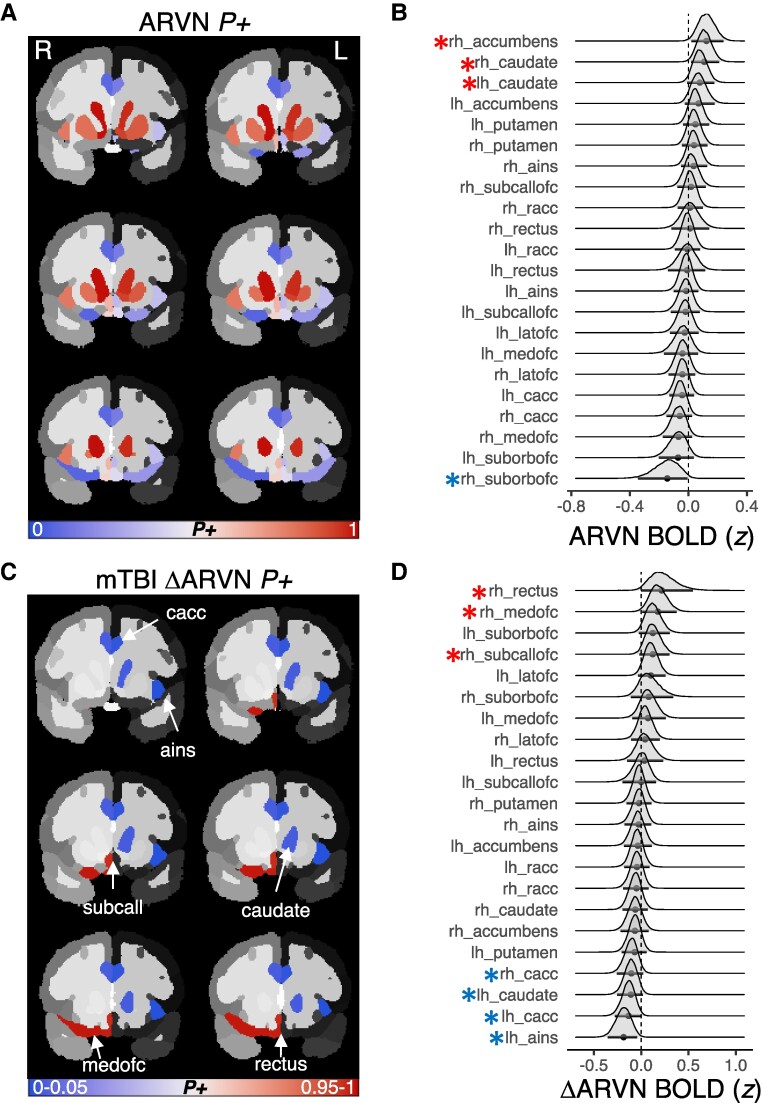
**Anticipate reward versus neutral (ARVN) contrast *ROI effect* and *mTBI change effect***. (**A**) ARVN BOLD contrast map depicting the probability of posterior samples above 0 (*P+*) by ROI, unthresholded to depict all ROIs included in the models. (**B**) Posterior densities of ARVN BOLD responses at each ROI. (**C**) mTBI-related change in ARVN BOLD *P+* values plotted by ROI, thresholded according to our *a priori* alpha level (*P+<*0.05, or >0.95), and (**D**) the mTBI-related change in ARVN posterior density plot at each ROI. Note: the red asterisks indicate 95% of posterior samples were above 0 (positive BOLD in **B**, or greater activation post-mTBI in **D**), and blue indicate only 5% were above 0 (negative BOLD in **B**, or reduced activation post-mTBI in **D**).

*Anticipate reward versus neutral.* The *ROI effects* model demonstrated statistically credible evidence for a positive BOLD signal during reward anticipation relative to neutral in right accumbens (Table 2), and bilateral caudate (Table 2; Fig. 3A and B). Credible evidence for negative reward anticipation BOLD was observed in right suborbital orbitofrontal cortex (Table 2; Fig. 3A and B). Looking at the change between baseline and follow-up, *Model 3* did not provide credible evidence for a reliable *ROI change effect* in any ROIs. Yet, it did provide evidence for a negative *mTBI change effect* on salience network recruitment across visits in both anterior insula (Table 2) and caudal ACC (Table 2; Fig. 3C and D). mTBI also appeared to induce greater recruitment of several orbitofrontal cortex (OFC) ROIs across study visits relative to controls (Table 2; Fig. 3C and D).

**Table 2:** Summary of statistically credible *ROI effects*, *ROI change effects*, and *mTBI change effects* from the series of fMRI-related Bayesian hierarchical models on reward trials

Contrast	ROI	Median	P+	95%-HDI	
				Lower	Upper
Reward—Anticipate reward versus neutral					
ROI effect	rh_suborbofc	−0.145	0.0187	−0.308	−0.0243
	**lh_caudate**	**0.0778**	**0.959**	**0.00437**	**0.159**
	**rh_caudate**	**0.108**	**0.991**	**0.031**	**0.193**
	**rh_accumbens**	**0.123**	**0.991**	**0.0352**	**0.221**
mTBI change	lh_ains	−0.186	0.0046	−0.325	−0.0627
	lh_cacc	−0.133	0.0174	−0.255	−0.0267
	lh_caudate	−0.109	0.046	−0.229	−0.00261
	rh_cacc	−0.105	0.0483	−0.23	−0.000938
	**rh_subcallofc**	**0.123**	**0.953**	**0.00186**	**0.268**
	**rh_medofc**	**0.176**	**0.983**	**0.0379**	**0.34**
	**rh_rectus**	**0.217**	**0.975**	**0.0289**	**0.484**
Reward—Anticipate large versus small reward					
ROI effect	rh_rectus	−0.32856	0.0022	−0.55873	−0.12625
	lh_rectus	−0.25696	0.001175	−0.42116	−0.11326
	lh_medofc	−0.17725	0.00745	−0.30101	−0.05674
	lh_cacc	−0.13023	0.016925	−0.23332	−0.03107
	**rh_accumbens**	**0.125356**	**0.96425**	**0.011227**	**0.245868**
	**lh_caudate**	**0.151815**	**0.99315**	**0.050329**	**0.256885**
	**rh_caudate**	**0.164029**	**0.996725**	**0.06493**	**0.268676**
	**lh_accumbens**	**0.187935**	**0.997225**	**0.073831**	**0.307179**
Reward—Positive versus negative feedback (gain)					
ROI effect	rh_ains	−0.24172	0.00005	−0.34584	−0.14111
	lh_ains	−0.22083	0	−0.31631	−0.1279
	rh_cacc	−0.21733	0.0001	−0.31596	−0.12184
	lh_cacc	−0.19728	0.0004	−0.29479	−0.10344
	rh_latofc	−0.10451	0.042	−0.20685	−0.00469
	**lh_putamen**	**0.121071**	**0.9767**	**0.021248**	**0.221885**
	**rh_putamen**	**0.150896**	**0.99465**	**0.053557**	**0.249781**
	**rh_accumbens**	**0.176644**	**0.99715**	**0.071721**	**0.279192**
	**lh_accumbens**	**0.200631**	**0.999**	**0.097295**	**0.305959**
	**rh_suborbofc**	**0.222446**	**0.98925**	**0.061299**	**0.406923**
	**lh_suborbofc**	**0.368922**	**1**	**0.218115**	**0.522665**
ROI change	lh_medofc	−0.0926	0.0468	−0.22	−0.00046
	lh_rectus	−0.231	0.0348	−0.615	−0.00446

***P+*** refers to the proportion of posterior samples above 0. In line with neuroimaging figure conventions—plain font indicates evidence for a negative effect (***P+***<0.05), and bold indicates evidence for a positive effect (***P+***>0.95). Dependent variables for all models were z-scored to aid cross-model interpretation.

##### Anticipate large versus small reward

We observed evidence that the same ROIs that demonstrated greater BOLD activation during reward anticipation relative to neutral are particularly enhanced during anticipation of a large versus a small reward. Specifically, the *ROI effects* model on the anticipated high versus low reward contrast evidenced a positive BOLD response to large versus small reward anticipation in the nucleus accumbens and caudate (Table 2). In contrast, medial OFC and caudal ACC demonstrated unique negative BOLD responses during anticipation of a large versus small reward (Table 2). *Model 5* did not provide credible evidence for a reliable *ROI change effect*, nor was there any evidence for an *mTBI change effect* on this contrast.

##### Positive feedback versus neutral feedback (gain)

In the *ROI effects* model, reward relative to nonreward feedback was associated with positive BOLD response in suborbital OFC, accumbens and putamen (Table 2), alongside deactivation of the salience network (anterior insula and caudal ACC ROIs) and lateral OFC (Table 2). *Model 7* did provide credible evidence for an *ROI change effect* in medial orbitofrontal cortex and gyrus rectus (Table 2), but there was no evidence of a statistically credible *mTBI change effect* at any ROI.

##### Punishment-related fMRI contrasts

**Figure 4:**
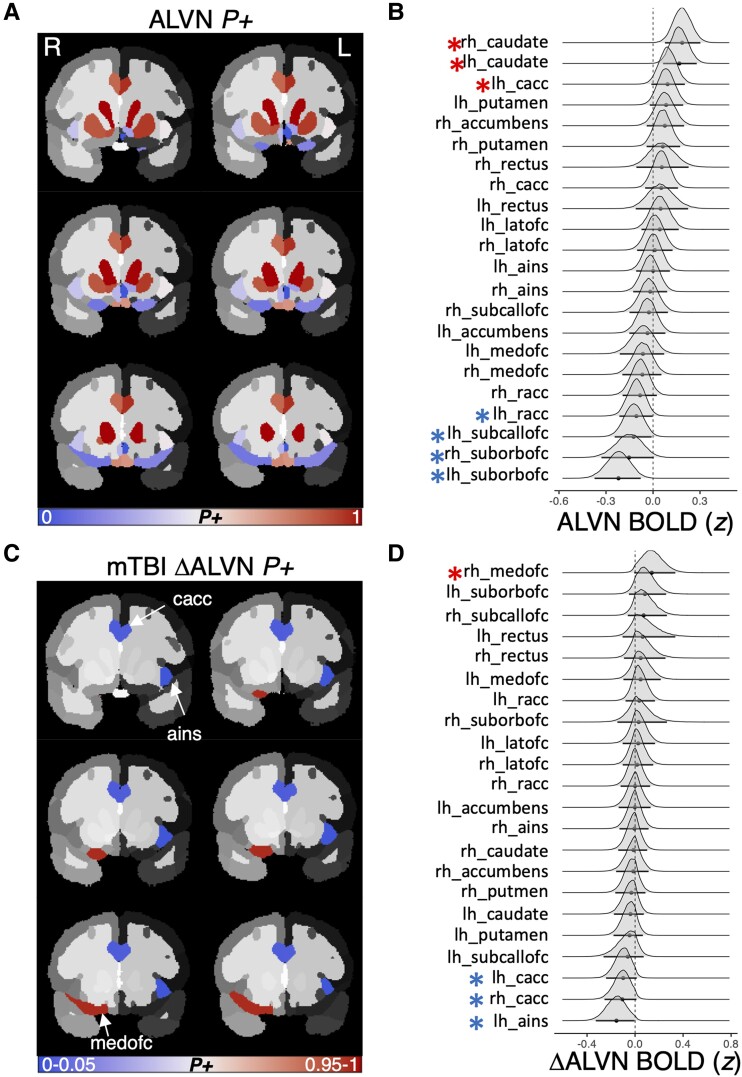
**Anticipate loss versus neutral (ALVN) contrast *ROI effect* and *mTBI change effect.*** (**A**) ALVN BOLD contrast map depicting the probability of posterior samples above 0 (*P+*) by ROI, unthresholded to depict all ROIs included in the models. (**B**) Posterior densities of ALVN BOLD responses at each ROI. (**C**) mTBI-related change in ALVN BOLD *P+* values plotted by ROI, thresholded according to our *a priori* alpha level (*P+<*0.05, or >0.95), and (**D**) the mTBI-related change in ALVN posterior density plot at each ROI. Note: the red asterisks indicate 95% of posterior samples were above 0 (positive BOLD in **B**, or greater activation post-mTBI in **D**), and blue indicate only 5% were above 0 (negative BOLD in **B**, or reduced activation post-mTBI in **D**).

*Anticipate loss versus neutral. O*ur *ROI effects* model on loss anticipation relative to neutral provided credible evidence for positive BOLD response in dorsal striatum (caudate) and caudal ACC (Table 3; Fig. 4A and B), as well as deactivation of rostral ACC and suborbital and subcallosal OFC (Table 3; Fig. 4A and B). *Model 4* did not provide evidence for an *ROI change effect* on BOLD recruitment during loss anticipation, however, it did indicate that—analogous to the anticipated reward versus neutral contrast—anterior insula and caudal ACC demonstrated a negative *mTBI change effect*, whereas medial OFC demonstrated a positive *mTBI change effect* across study visits (Table 3; Fig. 4C and D).

**Table 3:** Summary of statistically credible *ROI effects*, *ROI change effects*, and *mTBI change effects* from the series of fMRI-related Bayesian hierarchical models on loss trials

Contrast	ROI	Median	P+	95%-HDI	
				Lower	Upper
Punishment—Anticipate loss versus neutral					
ROI effect	lh_suborbofc	−0.22032	0.000625	−0.3483	−0.10121
	rh_suborbofc	−0.15309	0.0286	−0.29904	−0.02036
	lh_subcallofc	−0.12384	0.016875	−0.22462	−0.02658
	lh_racc	−0.10651	0.02185	−0.19733	−0.01961
	**lh_cacc**	**0**.**093058**	**0**.**96045**	**0**.**006013**	**0**.**184515**
	**lh_caudate**	**0**.**167776**	**0**.**99925**	**0**.**080708**	**0**.**261184**
	**rh_caudate**	**0**.**186047**	**0**.**999475**	**0**.**09447**	**0**.**283251**
mTBI change	lh_ains	−0.15483	0.0194	−0.29822	−0.0251
	rh_cacc	−0.10607	0.04365	−0.23016	−0.00265
	lh_cacc	−0.09886	0.04975	−0.2171	−0.0000398
	**rh_medofc**	**0**.**139193**	**0**.**964625**	**0**.**007776**	**0**.**302**
Punishment —Anticipate large versus small loss					
ROI effect	lh_cacc	−0.110	0.020	−0.213	−0.012
Punishment —Positive versus negative feedback (loss)					
ROI effect	lh_ains	−0.17463	0.00355	−0.28401	−0.06882
	rh_cacc	−0.16315	0.00495	−0.26861	−0.06207
	lh_cacc	−0.14959	0.0067	−0.25346	−0.05056
	rh_subcallofc	−0.1416	0.02525	−0.26521	−0.02221
	rh_ains	−0.13362	0.02	−0.24271	−0.02781
	lh_racc	−0.11884	0.0277	−0.2234	−0.01645
	**rh_suborbofc**	**0**.**165552**	**0**.**9592**	**0**.**008383**	**0**.**333089**
	**lh_accumbens**	**0**.**232329**	**0**.**9996**	**0**.**122908**	**0**.**344727**
	**rh_accumbens**	**0**.**236453**	**0**.**9996**	**0**.**123552**	**0**.**353041**
	**rh_putamen**	**0**.**261962**	**1**	**0**.**158366**	**0**.**367151**
	**lh_suborbofc**	**0**.**293564**	**0**.**99995**	**0**.**171641**	**0**.**419594**
	**lh_putamen**	**0**.**301455**	**1**	**0**.**19816**	**0**.**407749**

***P+*** refers to the proportion of posterior samples above 0. In line with neuroimaging figure conventions—plain font indicates evidence for a negative effect (***P+***<0.05), and bold indicates evidence for a positive effect (***P+***>0.95). Dependent variables for all models were z-scored to aid cross-model interpretation.

*Anticipate large versus small loss.* Akin to the anticipated loss versus neutral contrast, anticipation of a large versus small loss was associated with negative *ROI effects* in caudal ACC (Table 3). *Model 6* did not provide credible evidence for either an *ROI change effect* or an *mTBI change effect*.

*Positive feedback versus neutral feedback (avoid loss).* Receiving nonpunishment relative to loss was associated with positive BOLD response in dorsal striatum (putamen), accumbens and suborbital OFC (Table 3), alongside negative BOLD response in anterior insula, caudal *and* rostral ACC and subcallosal OFC (Table 3). *Model 8* did not provide credible evidence for either an *ROI change effect* or an *mTBI change effect*.

#### Are mTBI-related changes in motivational neural circuit recruitment associated with acquired depression?

The anticipated reward versus neutral contrast yielded credible evidence of mTBI-related change effects in several regions of medial frontal cortex, salience network and caudate (Fig. 3). We ran an additional model (*Model 9*) to determine if any of these ROI changes were associated with CBCL changes in depression across study visits within the mTBI group. This model did not reveal any credible associations between mTBI-related changes in BOLD responses to reward on the MID task (Fig. 3C and D) and mTBI-related changes in depression scores between study visits. We did not carry out *Models 10–11* as there was no statistically credible evidence of mTBI-related changes to correlate with depression on either the anticipate high versus low reward or receive reward versus neutral feedback MID contrasts.

### Exploratory analyses

#### Loss anticipation neural recruitment, depression and anxiety

The anticipated loss versus neutral contrast yielded credible evidence of *mTBI change effects* in several regions of medial frontal cortex, salience network and caudate (Fig. 4). We ran exploratory models to determine whether any of these ROI changes were associated with changes in depression or anxiety across study visits within the mTBI group. None of the ROIs that demonstrated statistically credible evidence for an *mTBI change effect* in the anticipated loss versus neutral contrast demonstrated credible associations with CBCL anxiety or depression scores. Notably, there was also no statistically credible evidence for a pre-post-mTBI increase in anxiety in the current sample (see Code section #12 of abcd_mTBI_depression_RR_2023_Stage2.html on osf.io/h5uba).

#### Cognition

##### mTBI-related changes in cognition

We determined whether a single mTBI event caused changes in NIH Toolbox cognition composite scores from the baseline to follow-up ABCD study visit. We also analyzed the association between time since injury and cognition change scores after a single mTBI. Notably, the participants included in our matched comparison did not complete some of the NIH Toolbox subtasks at follow-up (i.e. no participants completed working memory tasks or cognitive flexibility tasks at follow-up, and *N* = 4 CTRL and *N* = 3 mTBI were missing all Toolbox data at follow-up). Therefore, it was not possible to extract the standard overall cognition composite standard score at follow-up for our participants. We adjusted our approach to accommodate this by calculating an ‘average’ standard score across all the available cognition battery sub-tasks at both baseline and follow-up. On this measure, neither mTBI (*Baseline mean* = 104, *sem* = 1.21; *Follow-up mean* = 106, *sem* = 1.54; *difference d* = −0.35, *95%-HDI* = −0.79 to 0.14) nor the non-brain-injured control participants (*Baseline mean* = 103, *sem* = 1.74; *Follow-up mean* = 104, *sem* = 1.82; *difference d* = −0.18, *95%-HDI* = −0.69 to 0.33) demonstrated a statistically credible change in general cognitive functioning across visits. Additionally, a Bayesian multilevel model on cognition composite change scores did not indicate credible evidence that cognition changes in mTBI differed relative to controls (*b* = 0.35, *se* = 2.16, *95%-CI* = −3.87 to 4.60).

##### Time since mTBI and cognitive functioning

**Figure 5:**
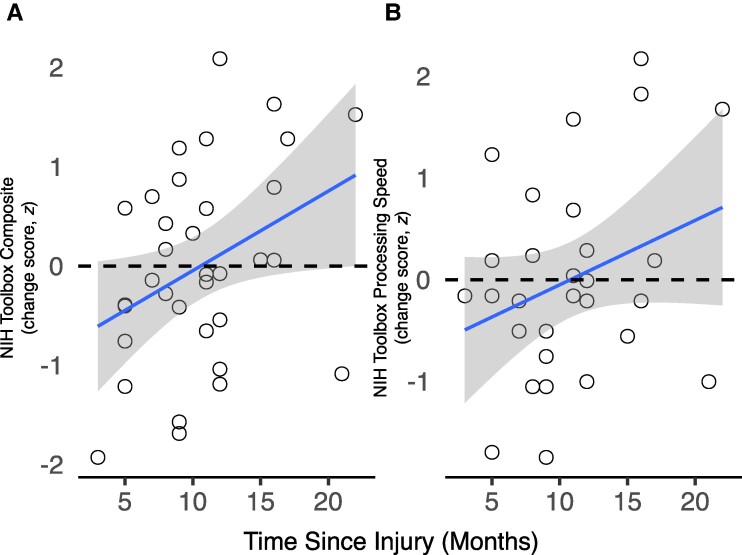
**Time since injury association with NIH toolbox cognition battery scores.** (**A**) There was a positive association between time since injury (in months) and average NIH-Toolbox Cognition battery change scores across baseline to follow-up study visits (*rho* = 0.35, *95%-HDI* = 0.02 to 0.64). (**B**) This association appeared to be primarily driven by a trend toward a specific association of time since injury and change scores on the pattern completion processing speed sub-task (*rho* = 0.28, *95%-HDI* = −0.08 to 0.62). These inferences involved Bayesian estimation-based analogues of the standard rank-ordered bivariate correlation test. Accordingly, the inferences included in the figure caption represent a Bayesian equivalent of Spearman’s rho for the effect size of the associations, as well as they 95% high density interval for the distribution of differences between those posteriors.

Notably, we did find credible evidence that the range of time since injury in the single mTBI group (3 to 23 months) was associated with a time-dependent pattern of post-injury cognitive deficit (i.e. cognition change scores were more negative after recent relative to older brain injuries; *rho* = 0.35, *95%-HDI* = 0.016 to 0.64; Fig. 5A). Given this observation, we explored which sub-task(s) of the NIH Toolbox that was available at both baseline and follow-up (i.e. processing speed, episodic memory, Flanker inhibition, oral reading and vocabulary) were likely driving this association. None of the individual subtasks were correlated with time since injury in mTBI on their own, but they all generally displayed the same positive signed correlation that we observed with the overall correlation. The only domain that demonstrated a statistically credible trend towards a relationship with time since injury was processing speed (*rho* = 0.28, *P* = 0.073; Fig. 5B; other subtask *rhos* −0.03 to 0.19, *p*s ≥ 0.15). Critically, these time-dependent changes in cognitive function post-mTBI were *not* associated with changes in post-mTBI depression (*rho* = 0.10, *P* = 0.284). Therefore, we *did* observe some modest evidence for a time-dependent change in cognitive functioning in participants who had experienced a more recent mTBI, which was likely driven by *less improvement* in processing speed from baseline to follow-up visits relative to participants with less proximal mTBI events. Notably, we ran additional exploratory correlations between change scores in NIH Toolbox processing speed with both (i) change scores in depression; and (ii) changes in MID task neural recruitment during reward and loss anticipation. None of these associations were statistically credible. Therefore, mTBI-related changes in processing speed and mTBI-related changes in MID task BOLD recruitment appear to represent statistically independent components of the mTBI phenotype in the current study.

## Discussion

This Registered Report evaluated whether mTBI causes depression and associated changes to motivational neural circuitry in late childhood/early adolescence using a prospective case-control design. Specifically—using the *ABCD study* cohort—we identified a subgroup of youth that had completed the same clinical, behavioural and neuroimaging battery both *before* and *after* a single mTBI event. We then compared those participants to an optimally-matched control group with no mTBI history before or in-between *ABCD* study visits. We found a striking lack of evidence for any mTBI-induced changes in depression in our preregistered analyses. We also failed to observe statistically credible evidence that multiple mTBIs or shorter time since injury predicted depression levels at follow-up—despite finding some modest evidence for a time since injury-dependent change in cognition (specifically, processing speed) in mTBI. In addition, despite evidence for mTBI-induced changes in medial prefrontal, orbitofrontal and cingulo-opercular ROIs during the anticipation of monetary incentives, none of these changes in BOLD fMRI outcomes were associated with changes in depression. Our findings suggest that chronic depression may *not* be a common post-mTBI symptom after a single mTBI event in late childhood/early adolescence.

According to existing large-scale studies using gold-standard self- and/or parent-report measures, approximately one-third of children and adolescents experience chronic post-concussion symptoms (PCS) after an mTBI event.^50,70,71,76,77^ To our knowledge, the current study is the first to examine whether depression—as well as other psychiatric (anxiety) and cognitive (NIH toolbox) symptoms of PCS—are elevated after an mTBI relative to a subject’s own preinjury scores on the same measures. The current data therefore indicate that parent-reported depression symptoms on the CBCL are not increased after an mTBI in the 9–14 year-old age range, which runs counter to several extant findings in the PCS field.

While existing studies have indicated elevated incidence of chronic mTBI sequelae in youth, the majority of those studies report on generalized measures of PCS. Relatively few studies have specifically looked at chronic depression following mTBI in youth. A recent review of this literature suggested that findings on this are mixed, with some data indicating substantial evidence for acquired depression following an mTBI in childhood or adolescence, while others find modest or null evidence for acquired depression after mTBI in this age group.^78^ Notably, prior studies that *do* observe clinically-significant acquired depression in youth have relied on retrospective cohort comparisons between mTBI and control groups 6 months or more *after* an mTBI event.^79‐81^ In contrast, a large prospective study by O’Conner *et al*. (2012) that took baseline depression measures in the subacute period (≈1-week) post-mTBI, and then collected the same measures during early (3-month) and late chronic (12- and 24-months) periods after an mTBI *did not* observe marked increases in the chronic incidence of depression after mTBI in youth.^82^ Depression may therefore be a clinically-significant yet low-incidence sequelae of mTBI in youth. It is possible that retrospective cohort studies of chronic mTBI in youth are confounded by incidental sampling enrichment of psychiatric sequelae. To be clear, youth and families dealing with chronic acquired depression after mTBI may be more likely to seek out advertised mTBI research studies relative to recovered mTBI. Prospective designs that do not find increased rates of new-onset depression in youth with chronic mTBI may therefore be less enriched for depressive symptoms *a priori* than analogous cohort comparison studies.

It is notable that prospective mTBI studies recruiting patients during the subacute period *have* found increased incidence of acquired depression in adults,^2^ and especially high depression in older relative to younger adults.^83^ For example, Brandt and colleagues examined the trajectory of depression outcomes in *N* = 30 adult mTBI patients (*M*_age_ = 28.96 ± 10.25) at multiple timepoints post-injury. They found that scores on the Beck Depression Inventory remained markedly high across the subacute (≈2-weeks)-to-early chronic (≈4-months) periods post-injury, relative to demographically-matched control participants.^2^ In line with this finding, a recent meta-analysis of chronic mTBI found a three-fold increase in depression risk after mTBI in adults relative to non-brain-injured controls, which did not decrease as a function of increased time since injury.^84^ Given the current findings, youth may have protective factors that make them more ‘resilient’ to chronic depression after mTBI relative to adults and geriatric populations. Psychological resilience—a trait associated with the ability to adapt to adversity—is a reliable positive predictor of recovery of function and is negatively associated with post-concussion symptoms after an mTBI in youth.^85,86^

At the molecular level, changes in gene expression in prefrontal circuitry across the lifespan overlap closely with genes that demonstrate aberrant transcription in depression in humans.^87^ This may represent an endogenous mechanism that increases idiopathic depression incidence in adulthood and old age relative to youth,^88^ which could in theory be compounded or accelerated by brain injury. Conversely, children and adolescents may be more resilient to new-onset depression after mTBI because they do not have this molecular predisposition to depression.

In contrast to the null findings for acquired depression, the current study did add to a growing corpus of evidence for aberrant functioning of medial prefrontal, orbitofrontal and cingulo-opercular (anterior cingulate cortex and anterior insula) in youth with mTBI (Fig. 3C and D; Fig. 4C and D). The current task-based fMRI results complement existing resting-state fMRI and magnetoencephalography studies that have observed intrinsic medial prefrontal, anterior cingulate and anterior insula aberrations after paediatric mTBI.^89,90^ Additionally, a recent well-powered dynamic resting-state functional connectivity study found that youth with mTBI spend less time occupying a brain state that comprises medial prefrontal, orbitofrontal, anterior insular and anterior cingulate cortex (as well as other temporopolar and lateral parietal regions) relative to non-brain-injured youth.^91^ Unfortunately, it is difficult to discern the functional impact(s) of these mTBI effects on static or dynamic brain network connectivity using resting-state imaging.

There is a relative dearth of *task*-based fMRI studies of mTBI in youth. The majority of existing studies in this field have focused on tasks that primarily involve executive functions, attentional orienting and sensorimotor control, e.g.^41,92^ In line with the current study, one study of inhibitory control in youth with mTBI observed blunted recruitment of anterior cingulate cortex in an emotional face variant of the go/no-go paradigm.^43^ Unfortunately, the MID task does not yield rich behavioural or decision-making variables to be examined in relation to task-related BOLD responses. This makes it difficult to infer the functional significance of our observed hyperactivation of medial prefrontal/orbitofrontal ROIs and hypoactivation of cingulo-opercular regions in mTBI. Additional task-based fMRI studies using novel approaches are needed to provide a more refined understanding of the neural computations in medial prefrontal, orbitofrontal and cingulo-opercular circuitry that are impacted by mTBI.

There are a few noteworthy limitations of the present Registered Report. First, the current study examined mTBI changes in depression and fMRI activity in the early-to-late chronic period, but it is possible that the study subjects would have demonstrated greater symptoms of acquired depression during the subacute period.^2^ Second, the *ABCD* study only collected the CBCL portion of the Achenbach System for Empirically-Based Assessment (ASEBA) battery but did not collect youth self-report or expert report data on depression or anxiety problems. It would have been useful to have multiple reporters (e.g. child self-report, teacher or clinician report) to determine whether parent-reports have decreased sensitivity to acquired depression or anxiety in youth after mTBI. Lastly, while the test–retest reliability and internal validity of the CBCL is known to be within the acceptable range in the current age range of 9–14 years, the psychometric properties of the MID task in this age range are not well-established.

Overall, the current study adds to existing prospective demonstrations suggesting that clinically-significant depressive symptoms are not a common chronic sequelae of a single *mild* TBI event in children and adolescents. As the *ABCD* cohort continues to age and future waves of data from the CBCL and MID task are released, researchers accessing these data should continue to monitor the effects of repeated injuries and moderate-to-severe injuries on depression. This will enable a well-powered examination of whether a more severe TBI burden may be more likely to impact psychiatric symptoms including depression in youth.

## Supplementary Material

fcae250_Supplementary_Data

## Data Availability

The data we used to carry out this Registered Report are available to approved users at the NIMH Data Archive (NDA; https://nda.nih.gov/abcd/). All code and sub-setted *ABCD* data required to recreate our results are available at http://dx.doi.org/10.15154/fv2d-y062 and the code is available for download at osf.io/h5uba.
