# Supplementary material for: Neural response to monetary incentives in acquired adolescent depression after mild traumatic brain injury: Stage 2 Registered Report
Source: Brain Commun. 2024 Sep 4;6(5):fcae250. doi: 10.1093/braincomms/fcae250 (PMC11371397; doi:10.1093/braincomms/fcae250)
Supplement: fcae250_Supplementary_Data [file fcae250_supplementary_data.pdf]

Title:

Neural response to monetary incentives in acquired adolescent depression after mild traumatic brain injury: Stage 1 Registered Report (protocol).

Author List:
Jeremy Hogeveen[1-2], Ethan M Campbell[1-2], Teagan S Mullins[1-2], Davin K. Quinn[3], Andrew R Mayer[1,3-5] & James F. Cavanagh[1-2]

Affiliations:
1. Department of Psychology, University of New Mexico, Albuquerque NM 87131 USA.
2. Psychology Clinical Neuroscience Center, University of New Mexico, Albuquerque NM 87131 USA.
3. Department of Psychiatry & Behavioral Sciences, University of New Mexico School of Medicine, Albuquerque NM 87131 USA.
4. Department of Neurology, University of New Mexico School of Medicine, Albuquerque NM 87131 USA.
5. The Mind Research Network/Lovelace Biomedical Research Institute, Pete & Nancy Domenici Hall, Albuquerque NM 87106 USA

*** Corresponding Author:** Jeremy Hogeveen, jhogeveen@unm.edu.

Abstract

Depression is a common neuropsychiatric consequence of traumatic brain injury (TBI) in adults. An emerging literature suggests depression may also occur at increased rates in adolescents following a TBI. Separately, spontaneous depression—i.e., not acquired via brain injury—has been linked to aberrant responses to the anticipation or receipt of rewards in motivational neural circuitry, which can be normalized following the use of pharmacologic or neurostimulation-based treatments for depression. Recent trials have begun to examine the efficacy of these same treatments for depression in patients with TBI, but it remains unknown whether there are common or distinct neural bases of spontaneous versus acquired depression. Determining whether motivational neural circuit responses to rewards are affected in acquired depression is therefore a vital step to i) providing empirical support for the translation of existing evidence-based treatments for depression to adolescents with acquired depression following TBI, and ii) providing further support for this prospective biomarker for identifying and monitoring treatment-induced change in depression. In this Registered Report, we will use the *Adolescent Brain Cognitive Development (ABCD)* consortium dataset to test the overarching research question that a disruption in functional neural responses to rewards is present in patients with acquired depression after TBI. Notably, *ABCD* provides an unparalleled opportunity to examine the trajectory of neuropsychiatric symptoms and behavior problems longitudinally within adolescent subjects—i.e., we can isolate TBI-specific variance independent from premorbid functioning. Specifically, here we focus on a case-control comparison between N=43 adolescents that experienced an mTBI between ABCD MRI visits, and N=43 well-matched controls (i.e., matched for demographics, as well as cognitive function and psychiatric symptoms at baseline). We first test the hypothesis that there will be significant increases in pre-post-injury depressive symptoms in mTBI patients relative to controls. Second, we hypothesize that mTBI will be associated with blunted recruitment of motivational neural circuitry during the anticipation or receipt of incentives on the ABCD study's Monetary Incentive Delay (MID) fMRI paradigm, specifically focusing on pre-post-injury changes in recruitment of striatum, anterior cingulate cortex (ACC), and anterior insula (aINS). Third, we will test the hypothesis that aberrant neural recruitment during the MID is associated with post-mTBI depressive symptoms. The proposed Registered Report will provide critical evidence for the shared vs. distinct functional neural mechanisms driving neurodevelopmental vs acquired depression. Regardless of our findings these data will provide critical evidence regarding the current standard of care for depression following mTBI in adolescence.

Introduction

Background and Motivation

Traumatic brain injury (TBI) is a significant global public health problem. Recent decades have seen increased recognition that even *mild* TBI (mTBI) has the potential to cause lasting functional difficulties that can impact the lives of patients and their families for weeks, months, or years post-injury (Centers for Disease Control and Prevention (CDC), 2003). Depression is one of the most common and persistent functional difficulties experienced by patients in the wake of an mTBI (Brandt et al., 2021; Holsinger et al., 2002; Silver et al., 2009), and is particularly common in patients with lesions to frontal, temporal, and parietal brain networks implicated in emotion and cognitive control (Koenigs et al., 2009; Mayer & Quinn, 2022). This has led to several recent clinical trials using antidepressant interventions (e.g. repetitive transcranial magnetic stimulation (rTMS), selective serotonin reuptake inhibitors (SSRIs), etc.) to remediate affective and cognitive sequelae of mTBI (Ashman et al., 2009; Barker-Collo et al., 2013; Olsson et al., 2023).

Existing studies of depression in mTBI are often limited by their ability to account for premorbid depressive symptoms. Specifically, the impact of mTBI on depression risk is most often studied cross-sectionally—i.e., comparisons between mTBI patients and control participants are used to provide evidence that depression risk is elevated after an mTBI. There is a central confound in such study designs: mTBI patients with elevated premorbid depression have been shown to report higher levels of lasting post-injury symptoms, which may cause an overall inflation of depression and other sequelae of mTBI in cross-sectional studies, independent of the physiological consequences of mTBI (Cicerone & Kalmar, 1997; Kirkwood et al., 2014; Max et al., 1997). Additionally, in cases of acquired depression after mTBI, it is unclear whether this occurs as a result of damage to the same neural circuits that underlie mood, anhedonia, or other depressive symptoms absent an mTBI event. Determining whether a single mTBI event can cause increases in depression—and whether acquired depression is driven by similar neural circuit mechanisms to depression without a prior brain injury—are both critical questions that need to be answered to inform evidence-based treatment of depression in mTBI patients.

Emerging results from the *Adolescent Brain Cognitive Development* [*ABCD*; (Volkow et al., 2018)] study provide an unparalleled opportunity to address these questions. The *ABCD* study is a large (*N*>10,000) longitudinal study of children and adolescents in the United States. *ABCD* participants and their parents began the study when participants were 9-10 years old, and a comprehensive battery of cognitive, behavioral, clinical, neuroimaging, and other assessments will continue to be administered on these subjects periodically until they reach young adulthood (19-20 years old). There are three specific reasons why *ABCD* can provide crucial insights into the questions addressed in the current Registered Report: 1) depressive symptoms and mTBI history interviews are administered at each study visit, meaning it is possible to determine whether mTBI events trigger lasting changes in depressive symptoms after controlling for premorbid functioning. 2) Multiple gold-standard clinical assays of

depression via parent-report are included in *ABCD*, providing superior assessment of multiple dimensions of the depressive phenotype (e.g. anhedonia, low mood, somatic complaints, etc.) relative to the majority of existing mTBI depression studies. And 3) the monetary incentive delay (MID) task—a functional neuroimaging tasks that has been reliably linked to depressive symptoms in adolescence (Fischer et al., 2019; Gotlib et al., 2010; Mori et al., 2016)—is being administered at each imaging visit in the *ABCD* study battery.

Monetary Incentive Delay (MID) **Task as a Functional Assay of Depression Mechanisms**

A promising functional neuroimaging task collected during imaging acquisition visits in *ABCD* that has been reliably linked to depression in adolescence is the *Monetary Incentive Delay (MID)* task. The *MID* is an fMRI task commonly used to probe the functional recruitment of motivational brain regions—including striatum and anterior cingulate cortex (ACC)—during the anticipation and receipt of incentives (Knutson et al., 2000). Incentives during the *MID* take the form of monetary gains or losses, each contrasted with a neutral nonreward control condition. This has relevance for depression given the consistent finding that differential behavioral and neural sensitivity to rewards and punishments has been reliably implicated in depression (Henriques et al., 1994; Henriques & Davidson, 2000; Knutson et al., 2008; Pizzagalli et al., 2008, 2009). In particular, existing *MID* fMRI studies have identified i) aberrant ACC and ventral striatal (nucleus accumbens, NAcc) recruitment during incentive anticipation (Hägele et al., 2015; Knutson et al., 2008; Stoy et al., 2012), ii) decreased recruitment of anterior insula (aINS) and brainstem during reward anticipation (Wakatsuki et al., 2022), iii) blunted recruitment of both NAcc and dorsal (caudate) striatum in response to rewarding feedback (Pizzagalli et al., 2009), and iv) decreased caudate-ACC functional connectivity in response to rewarding feedback (Admon et al., 2015) in depressed patients. Recruitment of these motivational and prefrontal brain regions in the *MID* is normalized in remitted depression and patients receiving stable doses of escitalopram (serotonin reuptake inhibitor) or amisulpride (dopamine receptor antagonist) (Admon et al., 2015, 2017; Dichter et al., 2012; Schiller et al., 2013; Stoy et al., 2012).

Whereas existing *MID* fMRI studies have primarily recruited adult participants, a key reason this task was included in the *ABCD* protocol is its demonstrated potential to probe the same motivational neural circuitry in adolescent and adult participants. Activation of basal ganglia nuclei (NAcc, caudate, globus pallidus, and putamen), ACC, aINS, and ventromedial prefrontal cortex has been reliably observed in adolescent participants during reward anticipation and receipt in the *MID* task (Cao et al., 2019; Lamm et al., 2014; Schneider et al., 2012; Silverman et al., 2014). Similar to adult participants, there is strong evidence that recruitment of striatum, aINS, ACC, and prefrontal cortex may be aberrant in adolescents with clinically-significant depression and those with an increased familial risk for developing depression in adulthood (Gotlib et al., 2010; Mori et al., 2016).

In sum, the *MID* task is an established technique for probing the neural bases of diminished reward sensitivity in depression. Aberrant neural circuit recruitment in response to monetary incentives during fMRI is a viable concomitant marker of the depressive phenotype. In

this study, we will examine this measure for the first time in adolescents before and after a brain injury.

mTBI Studies Using the *Adolescent Brain Cognitive Development (ABCD)* Study

At each *ABCD* study visit, parents of study participants complete the Ohio State University TBI Identification interview, a well-validated approach for retroactively identifying the frequency and severity of prior brain injury events (Corrigan & Bogner, 2007). Notably, two early studies have examined the sequelae of mTBI in adolescents via *ABCD*. First, Lopez and colleagues demonstrated that mTBI causes behavioral and emotional problems in adolescents, but found little evidence that brain *structure* was mediating these effects (Lopez et al., 2022). Given that most mTBIs are *not* associated with observable abnormalities on standard T1w or T2w MRI scans—apart from cases of 'complicated mTBI' [cf., (Mayer et al., 2017)]—functional MRI assays like the *MID* may be more sensitive predictors of post-mTBI symptom severity than structural imaging. Second, Sheth and colleagues recently demonstrated evidence for increased internalizing and other behavioral problems in adolescents following an mTBI in *ABCD* participants (Sheth et al., 2022). Both of these studies provide additional impetus for the current Registered Report—they suggest that emotional symptoms are increased in adolescents following an mTBI. However, no existing studies have leveraged *ABCD* to determine whether motivational neural circuit recruitment assayed via functional MRI predicts depression symptoms following an mTBI in adolescence.

Aims of the Current Registered Report

The main objective of the current Registered Report is to determine whether functional recruitment of motivational brain regions is aberrant in adolescents with acquired depression after an mTBI. This will involve three specific hypotheses that we will test using a Pre-registered Study Design (osf.io/h5uba):

> **Hypothesis 1:** Chronic mTBI causes new-onset depression in adolescence. This hypothesis will be tested by examining pre-post-mTBI changes in parent-reported depressive symptoms in adolescents in the *ABCD* study.
>
> **Hypothesis 2:** mTBI causes aberrant recruitment of motivational brain regions during the anticipation and receipt of monetary incentives. This hypothesis will be tested by examining pre-post-mTBI changes in responses during both reward and loss conditions on the *MID* task in ACC, aINS, and striatum (especially: caudate and NAcc).
>
> **Hypothesis 3:** mTBI-related changes in motivational neural circuitry will be associated with depressive symptoms. Specifically, we hypothesize a negative association, whereby mTBI-related increases in depression symptoms are expected to be associated with mTBI-related decreases in BOLD recruitment during the anticipation and receipt of rewards in the *MID* task.

Exploratory Hypothesis 4—Repetitive mTBI Versus Single mTBI. The current Registered Report was primarily designed to assess the impact of a *single* mTBI event on adolescent depression and task-related fMRI responses to incentives. Prior studies have provided evidence that repetitive versus single mTBI events may be more likely to trigger lasting acquired depression and changes in neural recruitment (Dams-O'Connor et al., 2013; Fann et al., 2004; Mayer & Quinn, 2022). *N*=22 participants in the *ABCD* data release 4.0 (2021) experienced multiple mTBIs between study visits (*N*=21 experienced 2, *N*=1 experienced 3), and this group is matched with respect to our primary demographic, cognitive, and head motion matching parameters relative to the control and single mTBI groups. Therefore, to explore whether repeated mTBI events cause increased depression symptoms or aberrant neural recruitment relative to a single mTBI event—we will repeat our proposed series of analyses testing hypotheses 1-3 but directly contrasting mTBI participants with a single versus 2-3 mTBI events between study visits.

Methods

Participants

As of *ABCD* Data Release 4.0 (2021; http://dx.doi.org/10.15154/1523041), *N*=43 participants experienced an mTBI in between the baseline and 2-year MRI imaging acquisition visits (*N*=27 reported at year 1 follow-up, and *N*=16 at year 2). These visits were conducted at one of the 21 study sites across the United States (https://abcdstudy.org/study-sites/) with data collection taking place between 09/01/2016—10/15/2018. The *ABCD* study was designed and implemented by a large team of scientists who were careful to mitigatge potential sources of bias [summarized in: (Garavan, 2018)]. This subset from the larger *ABCD* pool was selected because they offer the unique opportunity to examine premorbid-to-post-mTBI changes in depressive symptoms and neural recruitment in adolescence. We will also pull a randomly selected subset of well-matched healthy control participants with no history of possible brain injury from *ABCD*. This will be executed using the software package *MatchIt* to select participants using a nearest neighbor approximation (Ho et al., 2011), matching for key demographic and cognitive variables. Specifically, mTBI participants will be matched to a randomly selected group of control participants with respect to: handedness, age, race, ethnicity, sex, NIH Toolbox cognition composite scores (at baseline visit), CBCL depression scores (at baseline visit), mean framewise displacement / head motion during the MID scans, and socioeconomic status (indexed using the Area Deprivation Index, ADI; (Kind et al., 2014)). Given this was limited to participants with available data for all matching variables, the final sample of mTBI and control participants to be included in our analysis is a case-control matched subset of the larger *ABCD* cohort including *N*=43 mTBI and *N*=43 control participants (**Table 1**).

Sample Size Considerations

As there are no prior studies that examine pre-post mTBI neural recruitment within-subjects in a task fMRI paradigm in adolescence, we were forced to compare the proposed sample size to

prior studies using cross-sectional comparisons at a single time-point. From this review of the literature, the proposed sample is over three times the size of existing studies (e.g. N=12 mTBI versus control in (Yang et al., 2012); N=8 versus 8 in (Newsome et al., 2008); and N=11 versus 11 in (Tlustos et al., 2011)). Also notably, our within-subjects design looking at pre-post injury changes in symptoms is by definition a more powerful design than these cross-sectional studies (Brysbaert, 2019). We are also using the MID task which yields robust and large effect size fMRI responses to reward, detectable in small samples (N=6 needed for 0.80 power; (Knutson & Heinz, 2015)). Finally, we are limited in terms of our proposed power as this is a secondary data analysis rather than a *de novo* data collection effort. Accordingly: Given the importance of the research question, uniqueness of the dataset (i.e., ability to look at pre-post mTBI change within-subjects), and the fact that our sample is more than 3*the size of existing cross-sectional studies in this field—it is clinically and scientifically imperative to carry out this work.

Procedure

Brain injury identification. The *ABCD* study uses the Ohio State Brain Injury identification method (Corrigan & Bogner, 2007), administered both at baseline and each follow-up visit. For follow-up visits, the parent or guardian of each *ABCD* participant is asked eight questions, each involving follow-up responses:

1. Since we last saw you on [previous interview date], has your child been hospitalized or treated in an emergency room following an injury to their head or neck?
    1.1. (If 7 = “Yes”) Was your child knocked out or did they lose consciousness (LOC)? If yes, how long?
    1.2. (If 7 = “Yes”) Was your child dazed or did they have a gap in their memory from the injury?
    1.3. (If 7 = “Yes”) How old was your child?

2. Since we last saw you on [previous interview date], has your child injured their head or neck in a car accident or from crashing some other moving vehicle like a bicycle, motorcycle, or ATV? (If yes, 2.1-2.3 same as 1.1-1.3)

3. Since we last saw you on [previous interview date], has your child injured their head or neck in a fall or from being hit by something? (For example, falling from a bike or horse, rollerblading, falling on ice, being hit by a rock). In the past year, has your child injured their head or neck playing sports or on the playground? (If yes, 3.1-3.3 same as 1.1-1.3)

4. Since we last saw you on [previous interview date], has your child injured their head or neck in a fight, from being hit by something, or from being shaken violently? In the past year, has your child been shot in the head? (If yes, 4.1-4.3 same as 1.1-1.3)

5. Since we last saw you on [previous interview date], has your child been nearby when an explosion or blast has occurred? (If yes, 5.1-5.3 same as 1.1-1.3)

6. Do you want to report any more injuries with loss of consciousness since we last saw you on [previous interview date]? (If yes, 6.1-6.3 same as 1.1-1.3)

7. Since we last saw you on [previous interview date], did your child experience a period of time when they experienced multiple, repeated impacts to the head? (e.g., abused, contact sports)?
   7.1. Was your child knocked out or did they lose consciousness (LOC)? If yes, how long?
   7.2. Was your child dazed or did they have a gap in their memory from the injury?
   7.3. At what age did these effects begin?
   7.4. At what age did these effects end?

8. The next question asks about concussions. A concussion is when a blow or jolt to the head causes problems such as headaches, dizziness, being dazed or confused, difficulty remembering or concentrating, vomiting, blurred vision, or being knocked out. Since we last saw you on [previous interview date], how many times did your child have a concussion from playing a sport or being physically active?
   8.1. (If 8 = 1) Approximately how many days of school did your child miss, if any, due to the effects of the concussion?
   8.2. (If 8 > 1) Thinking about the most serious concussion your child experienced since we last saw on you [previous interview date], approximately how many days of school did your child miss, if any, due to the effects of the concussion?

TBI severity from these data is then determined according to the following spectrum: i) improbable TBI (no TBI or TBI without loss of consciousness or memory loss), ii) possible mTBI (TBI without loss of consciousness, but with memory loss), iii) mTBI (TBI with ≤30 minute loss of consciousness), iv) moderate TBI (TBI with loss of consciousness 30 minutes > 24 hours), and v) severe TBI (TBI with loss of consciousness ≥ 24 hours). Participants were also classified as whether or not they experienced repeated TBI events. Given the aims of this Registered Report, we focused exclusively on participants with a classic mTBI (i.e., including loss of consciousness ≤ 30 minutes), and only a single isolated mTBI event between ABCD study visits.

Depression. Clinical depressive symptoms were assessed using a combination of the Child Behavior Checklist (*CBCL*) via the Achenbach System for Empirically-Based Assessment (Achenbach & Rescorla, 2001) and a computerized administration of the Kiddie Schedule for Affective Disorders and Schizophrenia diagnostic tool [*K-SADS*; (Kaufman et al., 1997)]. The CBCL is a 112-item parent-administered assay of a broad range of psychological and behavioral problems in youth. Given the hypotheses of the current study we focused on the DSM-oriented composite score for depressive symptoms from the *CBCL*, which assays symptoms of Major Depression and Dysthymia. Additionally, given our specific interest in reward processing aberrations in depression, we will also examine the impact of mTBI on present anhedonia ratings based on retrospective reports in the *K-SADS*. The *K-SADS* administered in the *ABCD* study comprises parent-report, which demonstrates high concordance with clinician interview versions of the *K-SADS* (Barch et al., 2018). We opted to

rely on parent-report as there are no research-reliable clinician assessments in *ABCD*, and parent-report tends to map better onto clinician ratings of internalizing in adolescent participants compared to self-report (Hogeveen et al., 2018). This is a limitation of the current design, and one that should be followed up in future prospective youth mTBI studies that include clinician or other expert (e.g. teacher) reports of internalizing and externalizing problems to examine concordance across reporters.

Monetary incentive delay (MID) task. The *MID* task is a widely used method for probing motivational neural circuit recruitment during the anticipation and receipt of positive (monetary gain) or negative (monetary loss) feedback [**Figure 1A**; (Cao et al., 2019; Knutson et al., 2000)]. In the *ABCD* study version of the *MID* task, each trial begins with a 2 second long incentive cue that indicates one of five trial types: "Win $0.20," "Win $5," "Lose $0.20," "Lose $5," and "$0." This is followed by a fixation cross that is jittered between 1.5 and 4 seconds to optimize blood oxygenation-level dependent (BOLD) signal deconvolution during incentive anticipation—i.e., the "anticipation event" in Figure 1A. After the anticipation phase, a target is presented for 150-500 milliseconds, and participants are required to press a button as quickly as possible before the target disappears. The duration of target events are titrated to ensure that subjects are able to press the button before it disappears on ≈60% of trials. Lastly, the target is followed by a feedback event that tells the participant whether they have successfully received the reward ("Win" conditions), avoided the loss ("Lose" conditions), or that neither a win or loss has occurred ("$0" neutral condition). There will be 6 key event-related BOLD contrasts of interest in the current study will be: 1-2) anticipated incentive versus neutral (i.e., win versus neutral, and loss versus neutral), 3-4) anticipated high versus low incentive (i.e., win high versus low, and loss high versus low), and 5-6) positive versus negative feedback (i.e., win versus no win, and no loss versus loss).

Magnetic resonance imaging (MRI). The *ABCD* MRI acquisition and analysis protocols are elaborated in considerable detail elsewhere (Casey et al., 2018; Hagler et al., 2019). Briefly, MRI acquisition is taking place at 21 sites across the United States on 3T MRI scanners. The *ABCD* imaging team has harmonized the image acquisition sequences across all site scanners, but regardless study site will be included as a random effect in all inferential analyses in this Registered Report. Youth in the study complete motion-compliance training prior to their MRI scanning sessions in a mock MRI environment, and additional Framewise Integrated Real-Time MRI Monitoring Software [FIRMM; (Dosenbach et al., 2017)] is being used to monitor head motion in real time, enabling the scanning team to correct motion online by providing verbal feedback to participants or to collect additional data (Hagler et al., 2019). A robust fMRI preprocessing and analysis pipeline that is developed and maintained by the *ABCD* Data Analysis and Informatics Center—the Multi-Modal Processing Stream (Hagler et al., 2019)—is applied to all *ABCD* study data. This stream includes extensive within-subjects head motion de-contamination, including standard realignment parameters, their derivatives, and censoring of high motion timepoints (Satterthwaite et al., 2013; Siegel et al., 2014). We also included mean framewise displacement as a matching parameter to eliminate the potential for between-group differences in head motion to confound our primary analyses (**Table 1**). This processing stream also includes quality control of each post-processed structural and functional MRI scan by a

team of trained research technicians (Hagler et al., 2019). All processed data and tabulated region of interest (ROI) based analysis results are then made publicly available via the National Institute for Mental Health (NIMH) Data Archive (NDA). Subcortical ROI estimates in the publicly-released tabulated imaging data are segmented using the standard *Freesurfer* aseg atlas (Fischl et al., 2002), and cortical estimates are parcellated using the two standard *Freesurfer* aparc atlases [Desikan: (Desikan et al., 2006); Destrieux: (Destrieux et al., 2010)]. In the current Registered Report we used the Destrieux atlas given its higher parcel count and potential for greater functional specificity. The ROIs we will focus in the current Report on will comprise ACC, aINS, and striatum (**Figure 1B-C**). There are some notable limitations of this type of ROI-based approach (e.g. restricted concordance between ROIs and actual cluster-level activation patterns not always robust; (Zhi et al. 2022)). But notably, ROI-based approaches have also been argued to boost model efficiency and demonstrate a reduced tendency to overrepresent large cortical parcels close to the surface relative to traditional whole brain familywise error corrected inferences—alongside greater sensitivity to smaller subcortical and midline brain regions that are the focus of the current analyses (Chen et al. 2019).

Data Analysis Plan

Data analysis will be conducted using a combination of *Python v.3* and *R v.4* programming languages, and all code will be provided as Supplemental Material online. The proposed analyses will therefore be possible to recreate for any researchers with authorization to download the *ABCD* data.

Analysis plan for hypothesis 1. We will test the hypothesis that a single mTBI event causes increased post-injury depressive symptoms in adolescents. This hypothesis will be tested in a series of two Bayesian Multilevel Models using *brms v.2*. The first model (*Model 1*) with use pre-post-mTBI changes in *CBCL* DSM-module depression scores as the outcome variable:

$$Y = CBCL\ depression\ after\ mTBI - CBCL\ depression\ at\ baseline$$

The Y response distribution in *Model 1* will be fit as a Student t-distribution with non-informative priors set at *mu*=0 and *sigma*=1. This is intended to accommodate extreme, potentially influential observations at the tail ends of the distribution better than a Gaussian alternative. The model will take the form:

$$Y \sim 1 + TBI + (1 \mid site{:}subject)$$

In other words, we will model pre-post-mTBI changes in *CBCL* depression as a function of the fixed effect of TBI (1 = yes, 0 = no). We will also fit the nested random effects of $subject$ within *ABCD* study $site$. In *Model 1*, a posterior distribution providing credible evidence that the TBI term is above 0 [i.e., median estimate and lower and upper bounds of the 95% high-density interval (HDI)>0] will provide evidence for the alternative hypothesis: That mTBI status significantly increases *CBCL* depression scores.

Model 2 will be identical to *Model 1*, except the Y variable will be pre-post-mTBI changes in *K-SADS* ratings of anhedonia. This response variable will be coded as either 1 (symptom present after mTBI but not before) or 0 (either symptom stayed constant, or was reduced post-mTBI), and therefore the response distribution will be fit using default *brms* settings for non-informative priors in a binomial distribution. Again, if we observe statistically credible evidence (median and 95%-HDI>0) that the change in *K-SADS* anhedonic symptoms is positively moderated by mTBI status, this will provide additional evidence that single mTBI events cause increased levels of anhedonic depressive symptoms.

Analysis plan for hypothesis 2. The modeling approach for the *MID* task fMRI data will be similar to our approach for depression scores, but with some slight variations to enable us to simultaneously model all cortical and subcortical ROIs listed in **Figures 1B-C** [cf., (Chen et al., 2019; Hogeveen et al., 2022; Limbachia et al., 2021)]. Specifically, for each ROI we will compute the pre-post-mTBI difference score in BOLD responses for each *MID* task fMRI contrast, and fit separate Bayesian multilevel models for each contrast (6 fMRI Y variables):

Model 3: $Y1 = anticipate\ win\ versus\ neutral\ after\ mTBI - before\ mTBI$
Model 4: $Y2 = anticipate\ loss\ versus\ neutral\ after\ mTBI - before\ mTBI$
Model 5: $Y3 = anticipate\ high\ versus\ low\ win\ after\ mTBI - before\ mTBI$
Model 6: $Y4 = anticipate\ high\ versus\ low\ loss\ after\ mTBI - before\ mTBI$
Model 7: $Y5 = positive\ feedback\ win\ versus\ no\ win\ after\ mTBI - before\ mTBI$
Model 8: $Y6 = positive\ feedback\ no\ loss\ versus\ loss\ after\ mTBI - before\ mTBI$

Similar to *Model 1*, in *Models 3-8* the Y response distribution will be fit as a non-informative Student t-distribution centered at 0. These models will take the form:

$$Y \sim 1 + TBI + (TBI \mid ROI) + (1 \mid site : subject)$$

Given that we will have multiple observations for each study subject (i.e., multiple ROI estimates) in these models, in *Models 3-8* will fit the nested random effects of subject and study site. We will examine the posterior estimates for each ROI (i.e., each subcortical segment or cortical parcel in **Figure 1B-C**) in *Models 3-8* and determine how they are modulated by the fixed effect of mTBI status [i.e.,$(TBI \mid ROI)$] . Given that pre-post-injury changes in cognitive functioning are a common feature of the mTBI phenotype—and may inject unwanted random variation on the inferences in Models 3-8 (e.g., due to reduced vigilance during the task)—we will determine whether pre-post-mTBI changes in NIH Toolbox Cognition composite scores are associated with variables Y1-Y6 across the mTBI group. If so, these NIH Toolbox change scores will be included as random effects in Models 3-8.

Based on prior links to depression, we specifically hypothesize that the BOLD evoked responses during reward versus neutral anticipation, anticipation of high versus low rewards, and to the receipt of rewarding versus non-rewarding feedback will be negatively impacted by mTBI. In other words, we expect that some (primarily: NAcc and ACC) or all of the *a priori* subcortical and cortical ROIs will demonstrate **negative** modulation as a function of mTBI status

in *Model 3*, *Model 5*, and *Model 7*. Importantly, the main output from each *brms* model is a joint probability distribution across participants and ROIs, therefore multiple comparisons at the ROI-level are not appropriate or necessary (Gelman et al., 2012; Hogeveen et al., 2022; Limbachia et al., 2021). That said, given we will be examining a total of *N*=3 win-related contrasts across our primary depression-related fMRI models of interest, we will adjust the size of the posterior probability distribution that we would typically use to judge statistical credibility in Bayesian hierarchical models from 0.15 to 0.05 (i.e., 0.15 / 3 = 0.05). To be clear: For our critical models (9-11) posterior distributions where 95% of posterior MCMC estimates are above 0 will be used to indicate a statistically credible increase in BOLD activity as a function of mTBI, whereas those with 5% above 0 will indicate credible evidence for a decrease in BOLD activity as a function of mTBI).

Analysis plan for hypothesis 3. We will determine whether mTBI-related changes in neural recruitment during the anticipation and receipt of rewards is tied to mTBI-related changes in depression. This series of models (*Models 9-11*) will be performed on the same Y outcome variables as *Model 3, Model 5*, and *Model 7*, with the data filtered to only mTBI participants. These models will take the form:

Model 9: $Y1 \sim 1 + CBCL\ change + (CBCL\ change \mid ROI) + (1 \mid site : subject)$
Model 10: $Y3 \sim 1 + CBCL\ change + (CBCL\ change \mid ROI) + (1 \mid site : subject)$
Model 11: $Y5 \sim 1 + CBCL\ change + (CBCL\ change \mid ROI) + (1 \mid site : subject)$

Where *CBCL change* represents the pre-post-mTBI depression score change. We hypothesize that mTBI-related blunting of neural recruitment during the *MID* task will be associated with mTBI-related increases in depression. Therefore, any ROIs where the change in BOLD recruitment is negatively modulated as a function of changes in depression scores (i.e., blunted *MID* task BOLD signal post-mTBI associated with increased *CBCL Depression* scores post-mTBI) will provide credible evidence for hypothesis 3. For any significant effects of mTBI in models 9-11, we will also add *time since injury (in months)* as a potential covariate to determine whether the incremental validity of depression for predicting MID task neural recruitment remained significant in mTBI after covarying for the amount of time that elapsed between the brain injury event and the follow-up fMRI visit. Similar to Models 3, 5, and 7, for Models 9-11 statistical credibility will be evaluated based on whether the median and 95%-HDI estimates from the posterior distributions for each ROI are all *below* zero.

Robustness and convergence checks. Once our primary analysis plans are approved in this Stage 1 Report, we will conduct prior predictive checks on our primary outcome measures to determine whether the proposed Student's t non-informative priors are appropriate given the observed data, and will update model priors accordingly (e.g. to a uniform or Cauchy distribution depending on the direction indicated by our predictive checks). All brms models will use 4 Markov Chain Monte Carlo (mcmc) chains with 10,000 iterations per chain, and the convergence criterion will be R<1.1.

Exploratory analyses. We will conduct additional exploratory analyses to determine whether mTBI is associated with other psychiatric and cognitive sequelae in the current sample.

Other traumatic medical history. It is possible that the trauma and stress associated with a brain injury event—and not the physiological impacts of brain injury *per se*—might drive changes in neurodevelopment and depression that may confound the current study's central inferences. Therefore, we pulled an additional control measure from the *ABCD* dataset that will allow us to run a series of control analyses to mitigate this possibility: emergency room (ER) visits due to a broken bone. While other forms of early life adversity (e.g. food insecurity, emotional abuse, etc.) cause trauma and have been linked to internalizing in youth [e.g., (Centers for Disease Control and Prevention, 2021; Mendelson et al., 2008)], such experiences often persist across multiple timepoints and contexts. In contrast, hospital visits due to a broken bone versus an mTBI are both associated with a discrete event, locked to a specific time and context (e.g. a sporting injury), and are likely associated with similar degrees of subjective trauma. The broken bone group therefore represents an ideal comparison for comparing the impacts of a single, mild neurotrauma event *specifically* on internalizing. *N*=330 non-brain-injured control participants had a broken bone but no brain injury history, while *N*=3178 controls did not indicate a broken bone ER visit on their medical history. Therefore, we will replicate our primary analyses (i.e., Models 1-11) with the mTBI factor replaced by a binary predictor variable, and including *N*=330 participants with a broken bone in their medical history versus a matched group of *N*=330 control participants. For any statistically credible differences observed mTBI and controls in Models 1-11, we will conduct additional planned comparisons between the mTBI and broken bone groups.

Anxiety. We will repeat the analysis plan outlined for mTBI-related changes in *CBCL* depression scores, but including *CBCL* DSM-module anxiety scores. Akin to depression, acquired anxiety is also a clinically-significant consideration after an mTBI (Hsieh et al., 2012; Ponsford et al., 2016; Wood et al., 2014). Existing studies have linked anxiety to aberrant neural recruitment during the anticipation and receipt of incentives during the *MID* task, with studies consistently finding aberrant loss-evoked BOLD recruitment in individuals with various anxiety disorder phenotypes (Ernst et al., 2014; Held-Poschardt et al., 2018; Maresh et al., 2014). We will therefore hypothesize that ROIs where loss-related neural recruitment is impacted by mTBI in *Model 4*, *Model 6*, and *Model 8* will be associated with increased post-mTBI changes in anxiety symptoms.

Cognitive performance. While many patients experience lasting cognitive and emotional sequelae of mTBI, a significant degree of recovery appears to occur within 3-months to 1-year post-injury. Previous studies have suggested that a return to baseline depression levels after mTBI can be associated with concomitant resolution of cognitive deficits (Fann et al., 2001; Silver et al., 2009). Accordingly, we plan to analyze pre-post-mTBI changes in both fluid and crystallized cognitive functioning measures from the *NIH Toolbox* battery (Weintraub et al., 2013). If we observe credible evidence for hypothesis 1, we anticipate that mTBI subjects will demonstrate impaired performance on the *NIH Toolbox* cognition battery. Conversely, if chronic

mTBI patients in the current study do not demonstrate marked increases in depression, we hypothesize that they may also demonstrate similar performance on the *Toolbox* across visits.

Resting-state functional connectivity. Existing studies in the fMRI field suggest that changes in intrinsic brain architectures after mTBI can predict key post-injury neuropsychiatric sequelae (Hillary & Grafman, 2017; Hogeveen et al., 2021; Sharp et al., 2011; Siddiqi et al., 2023; Stephenson et al., 2020)**.** Again, the standard approach in this field is to compare fMRI outcomes between a group of mTBI and control participants at a single time point, and very few studies have managed to control for pre-injury effects. Given we have this opportunity with *ABCD*, in the current Report we will also conduct an exploratory analysis of resting-state functional connectivity between all *N*=20 cortical and subcortical ROIs (400 total network edges) and how pre-post mTBI changes in connectivity predict depression symptoms in mTBI and/or evoked BOLD responses during the MID task. This analysis will be conducted using a standard bivariate correlation approach with second-level covariates to determine the impact of mTBI status and depression symptoms in the CONN Toolbox (Whitfield-Gabrieli & Nieto-Castanon, 2012)**.**

Figures

Figure 1. (A) Schematic of the *ABCD* study's *Monetary Incentive Delay (MID)* task (Casey et al., 2018). Note that target duration is titrated based on individual reaction times to ensure ≈60% accuracy. **(B)** Subcortical regions-of-interest (ROIs) comprised the putamen, caudate, and nucleus accumbens, and **(C)** the cortical ROIs comprised medial and lateral parcels including lateral orbitofrontal cortex (lOFC), the short gyrus of the insula (anterior insula, aINS), caudal and rostral parcels of the anterior cingulate cortex (caudal ACC and rostral ACC), subcallosal gyrus (subCall), and both suborbital and medial sectors of OFC (subOrb and mOFC). Both subcortical segmentations and cortical parcellations in the *ABCD* study are generated using subject-specific surface reconstructions in the *Freesurfer* toolbox (Fischl et al., 2002).

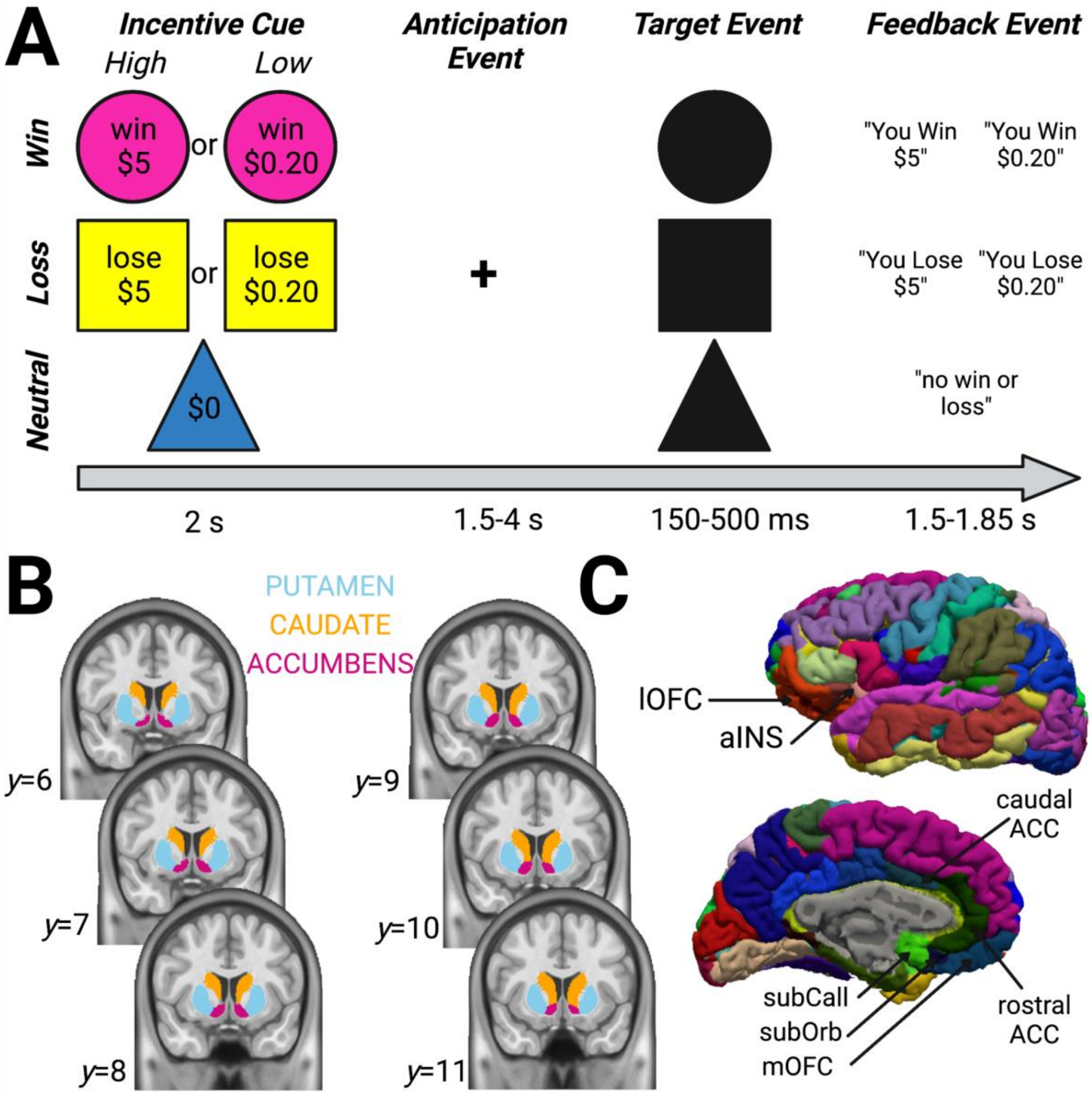

Tables

Table 1. Demographic and other key matching variables between mTBI and control participants.

Variable	**Control Participants**	**mTBI Participants**
N	43	43
Female (N)	19	14
Female (proportion)	0.44	0.33
Age (minimum, in months)	109	109
Age (SE, in months)	2.08	2.00
Caucasian (N)	35	35
Caucasian (proportion)	0.81	0.81
Hispanic (N)	6	9
Hispanic (proportion)	0.14	0.21
ADI (mean)	91.95	92.36
ADI (SE)	3.69	3.74
NIH Toolbox Composite (mean)	51.30	52.67
NIH Toolbox Composite (SE)	2.06	1.50
Head motion (Mean FD)	0.27	0.30
Head motion (SE FD)	0.04	0.04

Data Availability Statement

The data that we plan to use to carry out this Stage 1 Registered Report are available to approved users at the NIMH Data Archive (NDA; https://nda.nih.gov/abcd/). Upon study completion, all code and subsetted data required to recreate our results will be posted to a unique study link on NDA.

Acknowledgements

The Adolescent Brain Cognitive Development (*ABCD*) study is supported by the National Institutes of Health and additional federal partners under Award Numbers U01DA 041022, U01DA041028, U01DA041048, U01DA041089, U01DA041106, U01DA041117, U01DA041120, U01DA041134, U01DA041148, U01DA041156, U01DA041174, U24DA041123, U24DA041147, U01DA041093, and U01DA041025. ABCD data were accessed via https://nda.nih.gov/abcd/ under an approved data use certificate to the PI. While conducting this work, JH was supported through a center grant from the National Institute of General Medical Sciences (1P20GM109089), and independent grants from the National Institute on Alcohol Abuse and Alcoholism (R01AA030283) and the National Science Foundation BCD CogNeuro Program (CAREER 2237795).

References

Achenbach, T. M., & Rescorla, L. (2001). *Manual for the ASEBA school-age forms and profiles: An integrated system of multi-informant assessment.* Research Center for Children, Youth, and Families.

Admon, R., Kaiser, R. H., Dillon, D. G., Beltzer, M., Goer, F., Olson, D. P., Vitaliano, G., & Pizzagalli, D. A. (2017). Dopaminergic Enhancement of Striatal Response to Reward in Major Depression. *American Journal of Psychiatry*, *174*(4), 378–386. https://doi.org/10.1176/appi.ajp.2016.16010111

Admon, R., Nickerson, L. D., Dillon, D. G., Holmes, A. J., Bogdan, R., Kumar, P., Dougherty, D. D., Iosifescu, D. V., Mischoulon, D., Fava, M., & Pizzagalli, D. A. (2015). Dissociable cortico-striatal connectivity abnormalities in major depression in response to monetary gains and penalties. *Psychological Medicine*, *45*(1), 121–131. https://doi.org/10.1017/S0033291714001123

Ashman, T. A., Cantor, J. B., Gordon, W. A., Spielman, L., Flanagan, S., Ginsberg, A., Engmann, C., Egan, M., Ambrose, F., & Greenwald, B. (2009). A Randomized Controlled Trial of Sertraline for the Treatment of Depression in Persons With Traumatic Brain Injury. *Archives of Physical Medicine and Rehabilitation*, *90*(5), 733–740. https://doi.org/10.1016/j.apmr.2008.11.005

Barch, D. M., Albaugh, M. D., Avenevoli, S., Chang, L., Clark, D. B., Glantz, M. D., Hudziak, J. J., Jernigan, T. L., Tapert, S. F., Yurgelun-Todd, D., Alia-Klein, N., Potter, A. S., Paulus, M. P., Prouty, D., Zucker, R. A., & Sher, K. J. (2018). Demographic, physical and mental health assessments in the adolescent brain and cognitive development study: Rationale and description. *Developmental Cognitive Neuroscience*, *32*, 55–66. https://doi.org/10.1016/j.dcn.2017.10.010

Barker-Collo, S., Starkey, N., & Theadom, A. (2013). Treatment for depression following mild

traumatic brain injury in adults: A meta-analysis. *Brain Injury*, *27*(10), 1124–1133. https://doi.org/10.3109/02699052.2013.801513

Brandt, E., Wilson, J. K., Rieger, R. E., Gill, D., Mayer, A. R., & Cavanagh, J. F. (2021). Respiratory Sinus Arrhythmia Correlates With Depressive Symptoms Following Mild Traumatic Brain Injury. *Journal of Psychophysiology*, *35*(3), 139–151. https://doi.org/10.1027/0269-8803/a000268

Cao, Z., Bennett, M., Orr, C., Icke, I., Banaschewski, T., Barker, G. J., Bokde, A. L. W., Bromberg, U., Büchel, C., Quinlan, E. B., Desrivières, S., Flor, H., Frouin, V., Garavan, H., Gowland, P., Heinz, A., Ittermann, B., Martinot, J., Nees, F., … IMAGEN Consortium. (2019). Mapping adolescent reward anticipation, receipt, and prediction error during the monetary incentive delay task. *Human Brain Mapping*, *40*(1), 262–283. https://doi.org/10.1002/hbm.24370

Casey, B. J., Cannonier, T., Conley, M. I., Cohen, A. O., Barch, D. M., Heitzeg, M. M., Soules, M. E., Teslovich, T., Dellarco, D. V., Garavan, H., Orr, C. A., Wager, T. D., Banich, M. T., Speer, N. K., Sutherland, M. T., Riedel, M. C., Dick, A. S., Bjork, J. M., Thomas, K. M., … Dale, A. M. (2018). The Adolescent Brain Cognitive Development (ABCD) study: Imaging acquisition across 21 sites. *Developmental Cognitive Neuroscience*, *32*, 43–54. https://doi.org/10.1016/j.dcn.2018.03.001

Centers for Disease Control and Prevention (CDC). (2003). *Report to Congress on Mild Traumatic Brain Injury in the United States: Steps to Prevent a Serious Public Health Problem* [Data set]. US Department of Health & Human Services. https://doi.org/10.1037/e371602004-001

Chen, G., Xiao, Y., Taylor, P. A., Rajendra, J. K., Riggins, T., Geng, F., Redcay, E., & Cox, R. W. (2019). Handling Multiplicity in Neuroimaging Through Bayesian Lenses with Multilevel Modeling. *Neuroinformatics*, *17*(4), 515–545. https://doi.org/10.1007/s12021-018-9409-6

Cicerone, K. D., & Kalmar, K. (1997). Does premorbid depression influence post concussive symptoms and neuropsychological functioning? *Brain Injury*, *11*(9), 643–648. https://doi.org/10.1080/026990597123197

Corrigan, J. D., & Bogner, J. (2007). Initial Reliability and Validity of the Ohio State University TBI Identification Method. *Journal of Head Trauma Rehabilitation*, *22*(6), 318–329. https://doi.org/10.1097/01.HTR.0000300227.67748.77

Desikan, R. S., Ségonne, F., Fischl, B., Quinn, B. T., Dickerson, B. C., Blacker, D., Buckner, R. L., Dale, A. M., Maguire, R. P., Hyman, B. T., Albert, M. S., & Killiany, R. J. (2006). An automated labeling system for subdividing the human cerebral cortex on MRI scans into gyral based regions of interest. *NeuroImage*, *31*(3), 968–980. https://doi.org/10.1016/j.neuroimage.2006.01.021

Destrieux, C., Fischl, B., Dale, A., & Halgren, E. (2010). Automatic parcellation of human cortical gyri and sulci using standard anatomical nomenclature. *NeuroImage*, *53*(1), 1–15. https://doi.org/10.1016/j.neuroimage.2010.06.010

Dichter, G. S., Kozink, R. V., McClernon, F. J., & Smoski, M. J. (2012). Remitted major depression is characterized by reward network hyperactivation during reward anticipation and hypoactivation during reward outcomes. *Journal of Affective Disorders*, *136*(3), 1126–1134. https://doi.org/10.1016/j.jad.2011.09.048

Dosenbach, N. U. F., Koller, J. M., Earl, E. A., Miranda-Dominguez, O., Klein, R. L., Van, A. N., Snyder, A. Z., Nagel, B. J., Nigg, J. T., Nguyen, A. L., Wesevich, V., Greene, D. J., & Fair, D. A. (2017). Real-time motion analytics during brain MRI improve data quality and reduce costs. *NeuroImage*, *161*, 80–93. https://doi.org/10.1016/j.neuroimage.2017.08.025

Ernst, M., Plate, R. C., Carlisi, C. O., Gorodetsky, E., Goldman, D., & Pine, D. S. (2014). Loss aversion and 5HTT gene variants in adolescent anxiety. *Developmental Cognitive Neuroscience*, *8*, 77–85. https://doi.org/10.1016/j.dcn.2013.10.002

Fann, J. R., Uomoto, J. M., & Katon, W. J. (2001). Cognitive Improvement With Treatment of Depression Following Mild Traumatic Brain Injury. *Psychosomatics*, *42*(1), 48–54. https://doi.org/10.1176/appi.psy.42.1.48

Fischl, B., Salat, D. H., Busa, E., Albert, M., Dieterich, M., Haselgrove, C., van der Kouwe, A., Killiany, R., Kennedy, D., Klaveness, S., Montillo, A., Makris, N., Rosen, B., & Dale, A. M. (2002). Whole Brain Segmentation. *Neuron*, *33*(3), 341–355. https://doi.org/10.1016/S0896-6273(02)00569-X

Gotlib, I. H., Hamilton, J. P., Cooney, R. E., Singh, M. K., Henry, M. L., & Joormann, J. (2010). Neural Processing of Reward and Loss in Girls at Risk for Major Depression. *Archives of General Psychiatry*, *67*(4), 380. https://doi.org/10.1001/archgenpsychiatry.2010.13

Hägele, C., Schlagenhauf, F., Rapp, M., Sterzer, P., Beck, A., Bermpohl, F., Stoy, M., Ströhle, A., Wittchen, H.-U., Dolan, R. J., & Heinz, A. (2015). Dimensional psychiatry: Reward dysfunction and depressive mood across psychiatric disorders. *Psychopharmacology*, *232*(2), 331–341. https://doi.org/10.1007/s00213-014-3662-7

Hagler, D. J., Hatton, SeanN., Cornejo, M. D., Makowski, C., Fair, D. A., Dick, A. S., Sutherland, M. T., Casey, B. J., Barch, D. M., Harms, M. P., Watts, R., Bjork, J. M., Garavan, H. P., Hilmer, L., Pung, C. J., Sicat, C. S., Kuperman, J., Bartsch, H., Xue, F., … Dale, A. M. (2019). Image processing and analysis methods for the Adolescent Brain Cognitive Development Study. *NeuroImage*, 116091. https://doi.org/10.1016/j.neuroimage.2019.116091

Held-Poschardt, D., Sterzer, P., Schlagenhauf, F., Pehrs, C., Wittmann, A., Stoy, M., Hägele, C., Knutson, B., Heinz, A., & Ströhle, A. (2018). Reward and loss anticipation in panic disorder: An fMRI study. *Psychiatry Research: Neuroimaging*, *271*, 111–117. https://doi.org/10.1016/j.pscychresns.2017.11.005

Henriques, J. B., & Davidson, R. J. (2000). Decreased responsiveness to reward in depression. *Cognition & Emotion*, *14*(5), 711–724. https://doi.org/10.1080/02699930050117684

Henriques, J. B., Glowacki, J. M., & Davidson, R. J. (1994). Reward fails to alter response bias in depression. *Journal of Abnormal Psychology*, *103*(3), 460–466. https://doi.org/10.1037/0021-843X.103.3.460

Ho, D. E., Imai, K., King, G., & Stuart, E. A. (2011). **MatchIt**: Nonparametric Preprocessing for Parametric Causal Inference. *Journal of Statistical Software*, *42*(8). https://doi.org/10.18637/jss.v042.i08

Hogeveen, J., Mullins, T. S., Romero, J. D., Eversole, E., Rogge-Obando, K., Mayer, A. R., & Costa, V. D. (2022). The neurocomputational bases of explore-exploit decision-making. *Neuron*, *110*(11), 1869–1879.

Holsinger, T., Steffens, D. C., Phillips, C., Helms, M. J., Havlik, R. J., Breitner, J. C. S., Guralnik, J. M., & Plassman, B. L. (2002). Head Injury in Early Adulthood and the Lifetime Risk of Depression. *Archives of General Psychiatry*, *59*(1), 17. https://doi.org/10.1001/archpsyc.59.1.17

Hsieh, M.-Y., Ponsford, J., Wong, D., Schönberger, M., Taffe, J., & Mckay, A. (2012). Motivational interviewing and cognitive behaviour therapy for anxiety following traumatic brain injury: A pilot randomised controlled trial. *Neuropsychological Rehabilitation*, *22*(4), 585–608. https://doi.org/10.1080/09602011.2012.678860

Kaufman, J., Birmaher, B., Brent, D., Rao, U., Flynn, C., Moreci, P., Williamson, D., & Ryan, N. (1997). Schedule for Affective Disorders and Schizophrenia for School-Age Children-Present and Lifetime Version (K-SADS-PL): Initial Reliability and Validity Data. *Journal of the American Academy of Child & Adolescent Psychiatry*, *36*(7), 980–988. https://doi.org/10.1097/00004583-199707000-00021

Kirkwood, M. W., Peterson, R. L., Connery, A. K., Baker, D. A., & Grubenhoff, J. A. (2014). Postconcussive Symptom Exaggeration After Pediatric Mild Traumatic Brain Injury. *Pediatrics*, *133*(4), 643–650. https://doi.org/10.1542/peds.2013-3195

Knutson, B., Bhanji, J. P., Cooney, R. E., Atlas, L. Y., & Gotlib, I. H. (2008). Neural Responses

to Monetary Incentives in Major Depression. *Biological Psychiatry*, *63*(7), 686–692. https://doi.org/10.1016/j.biopsych.2007.07.023

Knutson, B., Westdorp, A., Kaiser, E., & Hommer, D. (2000). FMRI Visualization of Brain Activity during a Monetary Incentive Delay Task. *NeuroImage*, *12*(1), 20–27. https://doi.org/10.1006/nimg.2000.0593

Koenigs, M., Huey, E. D., Calamia, M., Raymont, V., Tranel, D., & Grafman, J. (2009). Distinct regions of prefrontal cortex mediate resistance and vulnerability to depression. *Journal of Neuroscience*, *28*, 12341–12348. https://doi.org/10.1523/JNEUROSCI.2324-08.2008.Distinct

Lamm, C., Benson, B. E., Guyer, A. E., Perez-Edgar, K., Fox, N. A., Pine, D. S., & Ernst, M. (2014). Longitudinal study of striatal activation to reward and loss anticipation from mid-adolescence into late adolescence/early adulthood. *Brain and Cognition*, *89*, 51–60. https://doi.org/10.1016/j.bandc.2013.12.003

Limbachia, C., Morrow, K., Khibovska, A., Meyer, C., Padmala, S., & Pessoa, L. (2021). Controllability over stressor decreases responses in key threat-related brain areas. *Communications Biology*, *4*(1), 42. https://doi.org/10.1038/s42003-020-01537-5

Lopez, D. A., Christensen, Z. P., Foxe, J. J., Ziemer, L. R., Nicklas, P. R., & Freedman, E. G. (2022). Association between mild traumatic brain injury, brain structure, and mental health outcomes in the Adolescent Brain Cognitive Development Study. *NeuroImage*, *263*, 119626. https://doi.org/10.1016/j.neuroimage.2022.119626

Maresh, E. L., Allen, J. P., & Coan, J. A. (2014). Increased default mode network activity in socially anxious individuals during reward processing. *Biology of Mood & Anxiety Disorders*, *4*(1), 7. https://doi.org/10.1186/2045-5380-4-7

Max, J. E., Robin, D. A., Lindgren, S. D., Smith, W. L., Sato, Y., Mattheis, P. J., Stierwalt, J. A. G., & Castillo, C. S. (1997). Traumatic Brain Injury in Children and Adolescents: Psychiatric Disorders at Two Years. *Journal of the American Academy of Child &*

Adolescent Psychiatry, *36*(9), 1278–1285. https://doi.org/10.1097/00004583-199709000-00021

Mayer, A. R., & Quinn, D. K. (2022). Neuroimaging Biomarkers of New-Onset Psychiatric Disorders Following Traumatic Brain Injury. *Biological Psychiatry*, *91*(5), 459–469. https://doi.org/10.1016/j.biopsych.2021.06.005

Mayer, A. R., Quinn, D. K., & Master, C. L. (2017). The spectrum of mild traumatic brain injury: A review. *Neurology*, *89*(6), 623–632. https://doi.org/10.1212/WNL.0000000000004214

Mori, A., Okamoto, Y., Okada, G., Takagaki, K., Jinnin, R., Takamura, M., Kobayakawa, M., & Yamawaki, S. (2016). Behavioral activation can normalize neural hypoactivation in subthreshold depression during a monetary incentive delay task. *Journal of Affective Disorders*, *189*, 254–262. https://doi.org/10.1016/j.jad.2015.09.036

Olsson, S. E., Singh, H., Kerr, M. S., Podlesh, Z., Chung, J., & Tjan, A. (2023). The role of transcranial magnetic stimulation in treating depression after traumatic brain injury. *Brain Stimulation*, *16*(2), 456–457. https://doi.org/10.1016/j.brs.2023.02.005

Pizzagalli, D. A., Holmes, A. J., Dillon, D. G., Goetz, E. L., Birk, J. L., Bogdan, R., Dougherty, D. D., Iosifescu, D. V., Rauch, S. L., & Fava, M. (2009). Reduced Caudate and Nucleus Accumbens Response to Rewards in Unmedicated Individuals With Major Depressive Disorder. *American Journal of Psychiatry*, *166*(6), 702–710. https://doi.org/10.1176/appi.ajp.2008.08081201

Pizzagalli, D. A., Iosifescu, D., Hallett, L. A., Ratner, K. G., & Fava, M. (2008). Reduced hedonic capacity in major depressive disorder: Evidence from a probabilistic reward task. *Journal of Psychiatric Research*, *43*(1), 76–87. https://doi.org/10.1016/j.jpsychires.2008.03.001

Ponsford, J., Lee, N. K., Wong, D., McKay, A., Haines, K., Alway, Y., Downing, M., Furtado, C., & O’Donnell, M. L. (2016). Efficacy of motivational interviewing and cognitive behavioral therapy for anxiety and depression symptoms following traumatic brain injury. *Psychological Medicine*, *46*(5), 1079–1090. https://doi.org/10.1017/S0033291715002640

Schiller, C. E., Minkel, J., Smoski, M. J., & Dichter, G. S. (2013). Remitted major depression is characterized by reduced prefrontal cortex reactivity to reward loss. *Journal of Affective Disorders*, *151*(2), 756–762. https://doi.org/10.1016/j.jad.2013.06.016

Schneider, S., Peters, J., Bromberg, U., Brassen, S., Miedl, S. F., Banaschewski, T., Barker, G. J., Conrod, P., Flor, H., Garavan, H., Heinz, A., Ittermann, B., Lathrop, M., Loth, E., Mann, K., & Martinot, J.-L. (2012). Risk Taking and the Adolescent Reward System: A Potential Common Link to Substance Abuse. *American Journal of Psychiatry*, *169*, 39–46.

Sheth, C., Huber, R. S., Renshaw, P. F., Yurgelun-Todd, D. A., & McGlade, E. C. (2022). Mild Traumatic Brain Injury and Behavior and Sleep Among 9- and 10-Year Old Children: Initial Findings From the Adolescent Brain Cognitive Development (ABCD) Study. *The Journal of Early Adolescence*, 027243162211175. https://doi.org/10.1177/02724316221117508

Silver, J. M., McAllister, T. W., & Arciniegas, D. B. (2009). Depression and Cognitive Complaints Following Mild Traumatic Brain Injury. *American Journal of Psychiatry*, *166*(6), 653–661. https://doi.org/10.1176/appi.ajp.2009.08111676

Silverman, M. H., Krueger, R. F., Iacono, W. G., Malone, S. M., Hunt, R. H., & Thomas, K. M. (2014). Quantifying familial influences on brain activation during the monetary incentive delay task: An adolescent monozygotic twin study. *Biological Psychology*, *103*, 7–14. https://doi.org/10.1016/j.biopsycho.2014.07.016

Stoy, M., Schlagenhauf, F., Sterzer, P., Bermpohl, F., Hägele, C., Suchotzki, K., Schmack, K., Wrase, J., Ricken, R., Knutson, B., Adli, M., Bauer, M., Heinz, A., & Ströhle, A. (2012). Hyporeactivity of ventral striatum towards incentive stimuli in unmedicated depressed patients normalizes after treatment with escitalopram. *Journal of Psychopharmacology*, *26*(5), 677–688. https://doi.org/10.1177/0269881111416686

Volkow, N. D., Koob, G. F., Croyle, R. T., Bianchi, D. W., Gordon, J. A., Koroshetz, W. J.,

Pérez-Stable, E. J., Riley, W. T., Bloch, M. H., Conway, K., Deeds, B. G., Dowling, G. J., Grant, S., Howlett, K. D., Matochik, J. A., Morgan, G. D., Murray, M. M., Noronha, A., Spong, C. Y., … Weiss, S. R. B. (2018). The conception of the ABCD study: From substance use to a broad NIH collaboration. *Developmental Cognitive Neuroscience*, *32*, 4–7. https://doi.org/10.1016/j.dcn.2017.10.002

Wakatsuki, Y., Ogura, Y., Hashimoto, N., Toyomaki, A., Miyamoto, T., Nakagawa, S., Inoue, T., & Kusumi, I. (2022). Subjects with bipolar disorder showed different reward system activation than subjects with major depressive disorder in the monetary incentive delay task. *Psychiatry and Clinical Neurosciences*, *76*(8), 393–400. https://doi.org/10.1111/pcn.13429

Weintraub, S., Dikmen, S. S., Heaton, R. K., Tulsky, D. S., Zelazo, P. D., Bauer, P. J., Carlozzi, N. E., Slotkin, J., Blitz, D., Wallner-Allen, K., Fox, N. A., Beaumont, J. L., Mungas, D., Nowinski, C. J., Richler, J., Deocampo, J. A., Anderson, J. E., Manly, J. J., Borosh, B., … Gershon, R. C. (2013). Cognition assessment using the NIH Toolbox. *Neurology*, *80*, S54–S64.

Wood, R. L. L., O'Hagan, G., Williams, C., McCabe, M., & Chadwick, N. (2014). Anxiety sensitivity and alexithymia as mediators of postconcussion syndrome following mild traumatic brain injury. *The Journal of Head Trauma Rehabilitation*, *29*, E9–E17. https://doi.org/10.1097/HTR.0b013e31827eabba

Brysbaert, M. (2019). How Many Participants Do We Have to Include in Properly Powered Experiments? A Tutorial of Power Analysis with Reference Tables. *Journal of Cognition*, *2*(1), 16.

Fischer, A. S., Ellwood-Lowe, M. E., Colich, N. L., Cichocki, A., Ho, T. C., & Gotlib, I. H. (2019). Reward-circuit biomarkers of risk and resilience in adolescent depression. *Journal of Affective Disorders*, *246*, 902–909.

Gelman, A., Hill, J., & Yajima, M. (2012). Why We (Usually) Don't Have to Worry About Multiple Comparisons. *Journal of Research on Educational Effectiveness*, *5*(2), 189–211.

Gotlib, I. H., Hamilton, J. P., Cooney, R. E., Singh, M. K., Henry, M. L., & Joormann, J. (2010). Neural processing of reward and loss in girls at risk for major depression. *Archives of General Psychiatry*, *67*(4), 380–387.

Hillary, F. G., & Grafman, J. H. (2017). Injured Brains and Adaptive Networks: The Benefits and Costs of Hyperconnectivity. *Trends in Cognitive Sciences*, *21*(5), 385–401.

Hogeveen, J., Aragon, D. F., Rogge-Obando, K., Campbell, R. A., Shuttleworth, C. W., Avila-Rieger, R. E., Yeo, R. A., Wilson, J. K., Fratzke, V., Brandt, E., Story-Remer, J., Gill, D., Mayer, A. R., Cavanagh, J. F., & Quinn, D. K. (2021). Ventromedial Prefrontal-Anterior Cingulate Hyperconnectivity and Resilience to Apathy in Traumatic Brain Injury. *Journal of Neurotrauma*, *38*(16), 2264–2274.

Hogeveen, J., Krug, M. K., Elliott, M. V., & Solomon, M. (2018). Insula-Retrosplenial Cortex Overconnectivity Increases Internalizing via Reduced Insight in Autism. *Biological Psychiatry*, *84*(4), 287–294.

Hogeveen, J., Mullins, T. S., Romero, J. D., Eversole, E., Rogge-Obando, K., Mayer, A. R., & Costa, V. D. (2022). The neurocomputational bases of explore-exploit decision-making. *Neuron*, *110*(11), 1869–1879.e5.

Kind, A. J. H., Jencks, S., Brock, J., Yu, M., Bartels, C., Ehlenbach, W., Greenberg, C., & Smith, M. (2014). Neighborhood socioeconomic disadvantage and 30-day rehospitalization: a retrospective cohort study. *Annals of Internal Medicine*, *161*(11), 765–774.

Knutson, B., & Heinz, A. (2015). Probing psychiatric symptoms with the monetary incentive delay task. *Biological Psychiatry*, *77*(5), 418–420.

Limbachia, C., Morrow, K., Khibovska, A., Meyer, C., Padmala, S., & Pessoa, L. (2021). Controllability over stressor decreases responses in key threat-related brain areas. *Communications Biology*, *4*(1), 42.

Mori, A., Okamoto, Y., Okada, G., Takagaki, K., Jinnin, R., Takamura, M., Kobayakawa, M., & Yamawaki, S. (2016). Behavioral activation can normalize neural hypoactivation in subthreshold depression during a monetary incentive delay task. *Journal of Affective Disorders*, *189*, 254–262.

Newsome, M. R., Steinberg, J. L., Scheibel, R. S., Troyanskaya, M., Chu, Z., Hanten, G., Lu, H., Lane, S., Lin, X., Hunter, J. V., Vasquez, C., Zientz, J., Li, X., Wilde, E. A., & Levin, H. S. (2008). Effects of traumatic brain injury on working memory-related brain activation in adolescents. *Neuropsychology*, *22*(4), 419–425.

Satterthwaite, T. D., Elliott, M. A., Gerraty, R. T., Ruparel, K., Loughead, J., Calkins, M. E., Eickhoff, S. B., Hakonarson, H., Gur, R. C., Gur, R. E., & Wolf, D. H. (2013). An improved framework for confound regression and filtering for control of motion artifact in the preprocessing of resting-state functional connectivity data. *NeuroImage*, *64*, 240–256.

Sharp, D. J., Beckmann, C. F., Greenwood, R., Kinnunen, K. M., Bonnelle, V., De Boissezon, X., Powell, J. H., Counsell, S. J., Patel, M. C., & Leech, R. (2011). Default mode network functional and structural connectivity after traumatic brain injury. *Brain: A Journal of Neurology*, *134*(Pt 8), 2233–2247.

Siddiqi, S. H., Kandala, S., Hacker, C. D., Bouchard, H., Leuthardt, E. C., Corbetta, M., Morey, R. A., & Brody, D. L. (2023). Precision functional MRI mapping reveals distinct connectivity patterns for depression associated with traumatic brain injury. *Science Translational Medicine*, *15*(703), eabn0441.

Siegel, J. S., Power, J. D., Dubis, J. W., Vogel, A. C., Church, J. A., Schlaggar, B. L., & Petersen, S. E. (2014). Statistical improvements in functional magnetic resonance imaging analyses produced by censoring high-motion data points. *Human Brain Mapping*, *35*(5), 1981–1996.

Stephenson, D. D., Meier, T. B., Pabbathi Reddy, S., Robertson-Benta, C. R., Hergert, D. C., Dodd, A. B., Shaff, N. A., Ling, J. M., Oglesbee, S. J., Campbell, R. A., Phillips, J. P.,

Sapien, R. E., & Mayer, A. R. (2020). Resting-State Power and Regional Connectivity After Pediatric Mild Traumatic Brain Injury. *Journal of Magnetic Resonance Imaging: JMRI*, *52*(6), 1701–1713.

Tlustos, S. J., Chiu, C.-Y. P., Walz, N. C., Holland, S. K., Bernard, L., & Wade, S. L. (2011). Neural correlates of interference control in adolescents with traumatic brain injury: functional magnetic resonance imaging study of the counting stroop task. *Journal of the International Neuropsychological Society: JINS*, *17*(1), 181–189.

Whitfield-Gabrieli, S., & Nieto-Castanon, A. (2012). Conn: a functional connectivity toolbox for correlated and anticorrelated brain networks. *Brain Connectivity*, *2*(3), 125–141.

Yang, Z., Yeo, R. A., Pena, A., Ling, J. M., Klimaj, S., Campbell, R., Doezema, D., & Mayer, A. R. (2012). An FMRI study of auditory orienting and inhibition of return in pediatric mild traumatic brain injury. *Journal of Neurotrauma*, *29*(12), 2124–2136.
